# Clinical Review, and Hospital Reports

**Published:** 1838-01-01

**Authors:** 


### Clinical Review.

OUR readers need not be told that we are most anxious to encourage Clinical Reports. The insulated facts of medicine may be observed in private practice, and may be communicated by private practitioners. But the masses of facts, as well as the great generalizations, can only be seen, and consequently can only be recorded, in public institutions for the sick.

We have never ceased to urge the medical officers of these institutions, to report what occurs within their walls. Some have responded to our call. The majority have not. Amongst those who have thus distinguished themselves by their zeal, and we will add their talent, we must place in the first rank the medical officers of Guy's Hospital. Honour to the metropolitan school which takes the lead in so noble a race, as to endeavour to diffuse useful knowledge through the ranks of our profession.

Some of the officers of the county hospitals have also evinced a readiness to come forward. For the proof of this we appeal to our present number.

The Report from the Kent and Canterbury Hospital is most creditable to the enthusiasm and the ability of a gentleman we are proud to call our friend—Dr. M'Divett. That Report we have inserted at full, and we are glad to be able to announce that it is the precursor of more.

We have but brief space at present for remarks. We would earnestly advise our provincial brethren to publish, in some form or other, regular clinical reports. Some should be statistic—some not; or, rather, there should, in each, be a combination of the two methods—statistic and particular. The former should, in our opinion, be subordinate to the latter. The professed statisticians expect too much from the application of the numerical method to diseases. It will assist us no doubt—it will tend to enlarge our views—it will correct misapprehensions—it will check the vanity of some, expose the artifice of others, and offer the best refutation to the pretensions of nostrum-mongers and to the credulity of their dupes—but it never will teach us to understand or to treat disease, as simple observation and rational experiment do. We make this protest against the abuse of statistics, because we are inclined to think that some persons expect too much from them, and that, having been long neglected, they are now in danger of being misapplied, and of giving birth to exaggerated notions of imaginary benefit.

In the reports of individual cases we would suggest the avoidance of prolix and diurnal details. The reports adapted for the ward-book are not adapted for the public. The sphere of duties of a clinical clerk and of a student is limited in a great degree to the narrow circle of one hospital. It is the merit of the former to relate each particular—of the latter to study it. But the professional reader of a journal has become acquainted with the mass of ordinary details, and with the effects of ordinary remedies, and neither time, nor inclination, nor utility will permit him to toil through timesome specifications of what he knows must happen. There is perhaps no point more difficult to hit than the juste-milieu between extreme indulgence in generalities or in details—but that *juste-milieu* the reporter should endeavour to hit.

It cannot be expected that the medical officers of hospitals should encounter the expense and the hazard of distinct publications. Their circulation would be small, and their cost would be great. We freely open our own columns to such Reports as are calculated to advance knowledge, and, as a kind of encouragement to the Reporters, we will give them the advantage of distinct type and a conspicuous place.

### Kent and Canterbury Hospital.

### Report of Medical Cases treated at the Kent and Canterbury Hospital during the Year 1836-7, By *John M'Divitt*, M.D. Physician to the Hospital.

The acting Medical Staff of the Kent and Canterbury Hospital consists of two physicians, four surgeons, and a resident house-surgeon. Dr. Carter, the senior physician, having resigned, I was elected his successor on the 9th of April, 1836. On the 22nd of the same month I took charge of such patients admitted under my predecessor as still remained on the books. They were 32 in number: viz. 9 in-patients and 23 out-patients.

Of the in-patients two were males, and 7 females, and their average residence in hospital had then amounted to 33 days nearly.

The out-patients consisted of 8 males and 15 females; and the average period of attendance had been 43 days.

From the date of my entering on office until the first of May, 1837* (being a period of little more than 12 months) I treated, exclusive of those already mentioned, 164 in—and 180 out-patients. In these numbers patients re-admitted for the same diseases are not reckoned twice, unless in cases where an interval of perfect health has existed between two distinct attacks.† Among the in-patients, there were only six re-admissions; and of these only one is counted twice. Nine out-patients were re-admitted; and of these four are reckoned twice, two as having remained in good health for a period of three months each after recovering from the first attack, and two as labouring the second time under different diseases. One of these last was admitted thrice, the second and third admissions being for a different disease from the first.

Fifteen out-patients admitted into hospital, and 40 in-patients made out, are numbered on the in-patient list only. In the case of the former, their average continuance as out-patients before admission into the wards had been 12 days. Of those "made out-patients" 12 still continue as such; the others attended as out-patients, for an average period of 42 days. To account for this prolonged attendance, it may be well to mention that I am in the habit of retaining the out-patient's names upon the books for a considerable period (often for three or four weeks) after their recovery has been established. This is done chiefly with a view to prevent, by keeping up the restraints of medical discipline, a speedy return to former pernicious habits,

---

* In consequence of a resolution of the governors of the hospital to enlarge the building by the addition of a second wing; and to make, at the same time, extensive alterations in the centre; no in-patients were admitted after the 11th of April; and as only patients of one sex could be accommodated during the continuance of the works, all the females were discharged before the 1st of May, at which time I happened to have only two male patients under my care. This, therefore, has seemed the best date for concluding my annual report; and, for the sake of convenience, I have adopted the same period for the out—as for the in-patients.

† I mention this fact, because it has been usual in the Canterbury Hospital, as in most other hospitals, to reckon every re-admission of a patient "discharged for overtime" as a new admission—a practice which for obvious reasons ought not to prevail.

which is so common a cause of dangerous relapses, particularly among the lower classes of society.

Of the in-patients, 62, and of the out-patients, 119, were from Canterbury or from some place within one mile of the hospital.

The out-patients were seen once a week;* and from observations made *during a part of the year*, I find that, while the weekly average on the books was 65, the average weekly attendance was 44, being about two-thirds of the whole. The average period of attendance was 59 days.

Of the in-patients the average residence in hospital was 52 days nearly.

The above particulars I have thought it necessary to premise before presenting the following summary of the results of treatment.

| | |
|---|---|
| In patients 164, of whom were cured | 71 |
| Relieved | 63 |
| Discharged without benefit | 7 |
| Died in hospital 5 } As out-patients 4 } | 9 |
| Remaining in hospital on 1st May, 1837 | 2 |
| Remaining as out-patients 1st May, 1837 | 12† |
| | 164 |

The above statement shows that the proportion of deaths occurring in the hospital was one in 32-8, or a fraction more than 3 per cent: but if we take into account the four deaths which took place among those "made out-patients," the proportion will be one in 18-2, or not quite 5½ per cent. In addition to these, I find, on strict enquiry, that of those discharged as "relieved" there died subsequently four; and of those who had received "no benefit" five; thus making 18 deaths in the whole. This gives a proportion of one death in rather more than 9 patients, or nearly 11 per cent. I shall by and by satisfactorily account for this apparently high rate of mortality. At present I may mention, that in nine of the fatal cases the disease was phthisis.

The cures in the cases of in-patients† amount to 43.2 per cent. and would have risen to a much greater number but for my extreme caution not to discharge as "cured" any patient, in whom there could be detected *the slightest remnant of organic disease*. Often, in affections of the chest, when the general symptoms appeared to warrant me in recording a "cure," the stethoscope discovered the existence of morbid changes, far beyond the power of our art to rectify, and calculated sooner or later to destroy life. It has frequently happened, besides, that a patient, though perfectly cured of the

* The rules of the hospital require that the out-patients shall be seen only once a week, but I am in the habit, during the intermediate days, of prescribing at my own house for all the more acute cases. The other medical officers, I believe, do the same.

† Of the 40 made out-patients, 28 were discharged prior to the 1st of May (vide page 177, note), the results of which cases are here distributed under the several heads of "cured," "relieved," &c. Twelve were cured, 11 were relieved, 4 died, and one was discharged having received no benefit. In a subsequent table (Table A.) the 12 here reckoned under the head of "remaining as out-patients," are in like manner distributed among the "cured," "relieved," &c.

‡ The number of out-patients cured amount to 46 per cent. Vide Table B.

disease for which he entered the hospital, has laboured under some other infirmity of long standing, and which has been, to a certain extent at least, the cause of the more recent affection. In every such case the patient has been discharged merely as "relieved," even when he has thought himself quite recovered, and has left the hospital with the intention of immediately resuming his employment. To some persons such strict caution may appear uncalled for and excessive, but I cannot help thinking it essential to the correctness, and consequently to the utility, of every record of cases. When a patient labouring under anasarca, as a consequence of diseased heart, has had all the effused serum removed by absorption, he is freed not from the disease, but merely from one of its symptoms: and yet nothing is more common than to report such a patient as "cured." Numerous instances of a similar kind might be adduced in exemplification of the point here insisted on.

But neither the proportion of deaths, nor that of cures, nor even both together, can be regarded as decisive of the amount of care and skill employed in the treatment; unless the age of the patients, the nature of the cases, and their duration previously to admission into hospital, be at the same time noted and considered. I shall therefore proceed to such a detail of the circumstances connected with these points, as, without being needlessly minute, may yet be sufficiently explanatory.

In manufacturing towns the poorer classes, when ill and unable to work, have no other resource than to apply for relief to some public charitable institution; and hence it happens that the hospitals, in such places, generally contain a tolerably large proportion of *acute* diseases. In agricultural districts, on the contrary, where the poor are less improvident, and, at the same time, are more attended to and assisted by their richer neighbours, medical relief is almost always obtained for a time at their own houses; and only the *more obstinate* and *chronic cases are sent into hospital.* Of the extent to which this is the case in Canterbury and its neighbourhood, some notion may be formed from the following tabular statement of the several periods during which, previously to admission, 148 of the in-patients treated by me had laboured under the same diseases for which they came under my care. The length of time during which the remaining 16 had been ill was neglected to be noticed when the cases were taken.

Of these 148 patients there had been ill—

| during 13 yrs. | 9 years | 8 years | 7 years | 5 years | 4 years | 3 to 4 years | 2 to 3 years | 1 to 2 years | 6 to 12 mths. | 3 to 6 mths. | 1 to 3 mths | under 1 mth. |
|---|---|---|---|---|---|---|---|---|---|---|---|---|
| 1 | 2 | 1 | 2 | 2 | 1 | 8 | 11 | 22 | 20 | 23 | 36 | 19 |

Their average duration of illness previously to admission was 487 days!

This has appeared to me so essential an element in every record of cases, that, with a view to still greater precision, I have inserted in the first of the tables which here follow, the average duration, prior to admission, *of each separate disease.* The mere *general average* indeed, can convey but very imperfect information, for a few cases of many years continuance will raise it very high, even when, as regards the remainder, it may be comparatively low.

In registering, on admission, the cases of the out-patients, this important point was not sufficiently attended to; and therefore in the table appropriated to them (Table B) no notice is taken of it.

### TABLE (A).

### IN-PATIENTS.

| NAME OF DISEASE. | Number of Patients affected with. | Average Age of same. | Average Number of Days ill before Admission. | Average Number of Days in Hospital. | Cured. | Relieved. | Discharged without Benefit. | Died in Hospital. | Date of Death, reckoning from Admission. | Died as Out-patients. | Known to have died after Discharge. |
|---|---|---|---|---|---|---|---|---|---|---|---|
| *Fevers.* | | years | days. | days. | | | | | day. | | |
| Simple Continued Fever.... | 1 | 22 | 21 | 49 | 1 | | | | | | |
| Typhoid Fever. .......... | 1 | 18 | 56 | 1 | | | | 1 | 2nd. | | |
| Chlorotic Fever ........ .. | 11 | 22 | 312 | 51 | 9 | 2 | | | | | |
| Gastro-Enteric Fever...... | 3 | 34 | 35 | 55 | 3 | | | | | | |
| Remittent Fever.......... | 7 | 10 | 165 | 42 | 5 | 1 | | 1 | 25th | | |
| Bilious Remittent Fever.... | 1 | 17 | 28 | 31 | 1 | | | | | | |
| Tertian Intermittent ...... | 7 | 26 | 107 | 31 | 7 | | | | | | |
| Quartan Intermittent...... | 1 | 13 | 150 | 42 | 1 | | | | | | |
| *Diseases of Head.* | | | | | | | | | | | |
| Hydrocephalus ........... | 1 | 31 | 63 | 42 | | 1 | | | | | |
| Hemiplegia ......... .... | 3 | 23 | 73 | 69 | 1 | 2 | | | | | |
| Other Affections of Head .. | 4 | 27 | 166 | 57 | 1 | 3 | | | | | |
| *Diseases of Thorax.* | | | | | | | | | | | |
| Pericarditis .............. | 4 | 11 | 48 | 49 | 3 | 1 | | | | | |
| Adherent Pericardium ..... | 3 | 20 | 242 | 49 | | 3 | | | | | |
| Diseased Heart ........... | 3 | 41 | 1230 | 51 | | 2 | | 1 | 21st. | | |
| Chronic Pleurisy.. ....... | 1 | 45 | 60 | 77 | | 1 | | | | | |
| Pleuro-Pneumonia ........ | 1 | 36 | 10 | 24 | 1 | | | | | | 1 |
| Empyema, &c............ | 1 | 37 | 730 | 63 | | 1 | | | | | |
| Pneumonia .............. | 1 | 36 | 50 | 42 | 1 | | | | | | 1 |
| Chronic Pneumonia ....... | 2 | 19 | 530 | 37 | | | 1 | 1 | 50th | | |
| Hæmoptysis .. .......... | 1 | 14 | 14 | 21 | 1 | | | | | | |
| Pulmonary Apoplexy...... | 1 | 19 | 90 | 13 | | | | 1 | 13th | | |
| Phthisis ...... ........ | 18 | 26 | 171 | 46 | | 11 | 3 | | | 4 | 5 |
| Spasmodic Asthma........ | 1 | 42 | 3295 | 21 | | 1 | | | | | |
| Acute Bronchitis.......... | 3 | 30 | 14 | 15 | 3 | | | | | | |
| *Diseases of Abdomen.* | | | | | | | | | | | |
| Chronic Peritonitis........ | 1 | 34 | 120 | 56 | | 1 | | | | | |
| Gastritis ....... ........ | 15 | 23 | 427 | 49 | 11 | 4 | | | | | |
| Gastrodynia....... ...... | 1 | 31 | 565 | 2 | | | 1 | | | | |
| Hæmorrhage from Stomach and Bowels .... | 1 | 23 | 730 | 56 | | | 1 | | | | 1 |
| Melæna, &c.............. | 1 | 59 | 60 | 63 | | | 1 | | | | |
| Dyspepsia ............... | 5 | 51 | 180 | 32 | 1 | 4 | | | | | |
| Intestinal Irritation ....... | 9 | 35 | 436 | 65 | 2 | 7 | | | | | |
| Dysentery .............. | 1 | 46 | 28 | 80 | | 1 | | | | | 1 |
| Chorea.................. | 1 | 11 | 42 | 70 | | 1 | | | | | |
| Hysteria ............... | 1 | 18 | 21 | 21 | 1 | | | | | | |
| Hysterical Paralysis ...... | 1 | 28 | 2920 | 101 | 1 | | | | | | |
| Chronic Hepatitis ........ | 1 | 21 | 545 | 84 | | 1 | | | | | |
| Jaundice, &c. ....... .... | 1 | 15 | 180 | 56 | | 1 | | | | | |

| | | | | | | | | | | | |
|---|---|---|---|---|---|---|---|---|---|---|---|
| Diseased Pancreas ....... | 1 | 59 | 180 | 53 | | 1 | | | | | |
| Diseased Kidneys ... ..... | 1 | 50 | 14 | 75 | 1 | | | | | | |
| Abdominal Aneurism...... | 1 | | | 35 | | 1 | | | | | |
| *Diseases of Pelvis.* | | | | | | | | | | | |
| Diseased Uterus ......... | 3 | 51 | 334 | 68 | | 3 | | | | | |
| Retentio-Mensium .... ... | 3 | 17 | | 48 | 3 | | | | | | |
| Diseased Bladder.......... | 1 | 70 | 1095 | 49 | | 1 | | | | | |
| *Spine.* | | | | | | | | | | | |
| Paraplegia .............. | 4 | 31 | 968 | 107 | 1 | 3 | | | | | |
| *Joints.* | | | | | | | | | | | |
| Acute Rheumatism ....... | 8 | 24 | 21 | 26 | 8 | | | | | | |
| Chronic Rheumatism...... | 10 | 35 | 313 | 81 | 4 | 6 | | | | | |
| *Muscular and Fibrous Parts.* | | | | | | | | | | | |
| Lumbago .. ............ | 5 | 49 | 765 | 37 | 2 | 3 | | | | | |
| Sciatica.................. | 1 | 42 | 120 | 105 | 1 | | | | | | |
| Rheumatic Periostitis...... | 1 | 26 | 730 | 74 | | 1 | | | | | |
| Syphilitic Pains .......... | 1 | 40 | 60 | 35 | 1 | | | | | | |
| *Thyroid Gland.* | | | | | | | | | | | |
| Bronchocele ............. | 1 | 14 | 1460 | 77 | 1 | | | | | | |
| *Eye.* | | | | | | | | | | | |
| Choroiditis .............. | 1 | 17 | 545 | 140 | | 1 | | | | | |
| *Skin.* | | | | | | | | | | | |
| Lepra Vulgaris ........... | 1 | 19 | 545 | 49 | | 1 | | | | | |
| Psoriasis Inveterata ....... | 1 | 16 | 4745 | 84 | 1 | | | | | | |
| Eczema Impetiginodes ..... | 1 | 28 | 8 | 95 | 1 | | | | | | |
| Total.... | 164 | | | | 78 | 70 | 7 | 5 | | 4 | 9 |

### TABLE (B).

OUT-PATIENTS.

| NAME OF DISEASE. | Number of Patients affected with. | Average Age of same. | Average Period of Attendance. | Cured. | Relieved. | Discharged without Benefit. | Discharged for Non-attendance. | Died. |
|---|---|---|---|---|---|---|---|---|
| *Fevers.* | | years | days. | | | | | |
| Chlorotic Fever ..................... | 11 | 18 | 68 | 8 | 2 | | 1 | |
| Fever from prolonged Lactation ........ | 3 | 27 | 39 | 2 | | | 1 | |
| Gastro-Enterite ..................... | 1 | 21 | 49 | 1 | | | | |
| Remittent Fever...... .............. | 9 | 6 | 66 | 6 | 2 | | 1 | |
| Tertian Ague ....................... | 3 | 25 | 21 | 3 | | | | |
| Quartan Ague........................ | 1 | 34 | 35 | 1 | | | | |
| *Diseases of Head.* | | | | | | | | |
| Hydrocephalus ...................... | 2 | 19 | 84 | 1 | 1 | | | |
| Epilepsy ...... .................... | 3 | 22 | 52 | 1 | 2 | | | |
| Other Affections of Head .............. | 8 | 42 | 63 | 3 | 2 | | 2 | 1 |

| *Diseases of Thorax and Air-Passages.* | | years | days. | | | | | |
|---|---|---|---|---|---|---|---|---|
| Diseased Heart | 8 | 46 | 70 | | 7 | 1 | | |
| Aneurism of Aorta | 1 | 57 | 84 | | 1 | | | |
| Chronic Pleurisy | 1 | 24 | 84 | | 1 | | | |
| Pneumonia, &c. | 1 | 18 | 84 | 1 | | | | |
| Hæmoptysis | 1 | 18 | 21 | 1 | | | | |
| Phthisis | 18 | 29 | 62 | | 8 | 1 | | 9 |
| Asthma | 4 | 49 | 82 | 1 | 3 | | | |
| Acute Bronchitis | 1 | 20 | 14 | 1 | | | | |
| Chronic Bronchitis | 10 | 33 | 75 | 5 | 4 | | | 1 |
| Simple Catarrh | 1 | 19 | 21 | 1 | | | | |
| Dry Catarrh | 1 | 76 | 14 | | | | 1 | |
| Laryngitis | 1 | 22 | 14 | 1 | | | | |
| Effects of Tight Lacing | 1 | 19 | 42 | 1 | | | | |
| *Diseases of Abdomen.* | | | | | | | | |
| Chronic Peritonitis | 2 | 37 | 73 | | 2 | | | |
| Gastritis | 3 | 26 | 57 | 3 | | | | |
| Gastrodynia | 1 | 53 | 77 | 1 | | | | |
| Dyspepsia | 12 | 44 | 62 | 1 | 10 | | 1 | |
| Intestinal Irritation | 4 | 13 | 56 | 4 | | | | |
| Tympanitis | 1 | 28 | 77 | 1 | | | | |
| Iliac Pain | 1 | 56 | 42 | | | 1 | | |
| Diarrhœa | 2 | 5 | 30 | 2 | | | | |
| Worms | 3 | 17 | 77 | 1 | 1 | 1 | | |
| Chorea | 1 | 13 | 84 | 1 | | | | |
| Hysteria | 4 | 28 | 73 | 3 | 1 | | | |
| Chronic Hepatitis | 2 | 36 | 84 | 2 | | | | |
| *Diseases of Pelvis* | | | | | | | | |
| Deranged Menstruation | 1 | 45 | 74 | 1 | | | | |
| Menorrhagia | 3 | 40 | 60 | 1 | 2 | | | |
| Diseased Uterus | 3 | 55 | 75 | | 3 | | | |
| *Diseases of Genitals.* | | | | | | | | |
| Leucorrhœa | 1 | 46 | 42 | 1 | | | | |
| Gonorrhœa | 1 | 21 | 84 | 1 | | | | |
| *Spine.* | | | | | | | | |
| Diseased Spine | 1 | 53 | 84 | | | 1 | | |
| *Joints.* | | | | | | | | |
| Acute Rheumatism | 1 | 4 | 11 | 1 | | | | |
| Chronic Rheumatism | 7 | 56 | 79 | 1 | 5 | | 1 | |
| *Muscular and Fibrous Parts.* | | | | | | | | |
| Lumbago | 2 | 32 | 45 | | 1 | | 1 | |
| *Thyroid Gland.* | | | | | | | | |
| Bronchocele | 1 | 15 | 84 | | 1 | | | |
| *Eye.* | | | | | | | | |
| Amaurosis | 1 | 29 | 21 | | | 1 | | |
| *Skin.* | | | | | | | | |
| Syphilis | 4 | 21 | 73 | 4 | | | | |
| Secondary Syphilis | 1 | 11 | 84 | | 1 | | | |
| Porrigo Lupinosa | 5 | 8 | 84 | 5 | | | | |
| Porrigo Decalvans | 1 | 13 | 84 | | 1 | | | |
| Ecthyma Scabies | 1 | 12 | 51 | 1 | | | | |
| Erythema Nodosum | 1 | 29 | 56 | 1 | | | | |
| Lepra Vulgaris | 4 | 10 | 39 | 3 | 1 | | | |
| *Diseases of Nerves.* | | | | | | | | |
| Paralysis of Arm | 1 | 15 | 56 | | 1 | | | |
| Neuralgia | 6 | 38 | 57 | 5 | 1 | | | |
| Simple Debility | 7 | 51 | 84 | 2 | 2 | 2 | 1 | |
| Senile Dissolution | 1 | 78 | 35 | | | 1 | | |
| Total.... | 180 | | | 84 | 66 | 9 | 10 | 11 |

It was my intention, when I began to draw up this report, to attempt a pathological classification of the diseases of which it treats; but I soon became satisfied, that, in the present state of our knowledge, such an attempt must prove unsuccessful. Neither the healthy nor the morbid structure of our frames is yet sufficiently understood to enable us to determine accurately the seat, much less the essential nature, of each particular disease. The investigations of modern anatomists, it is true, have done much towards the attainment of these objects by making us acquainted with the special properties of the several tissues, and the peculiar changes which they individually undergo in disease: and this, I apprehend, is the course of investigation, by which, if by any, a precise and truly natural classification of diseases may be arrived at. Great, however, and manifold are the difficulties which must be encountered by those who undertake such a task. For even supposing the primary obstacle (namely, our imperfect knowledge of the tissues, whether in their healthy or diseased state) removed, yet many others of no small magnitude would still continue to obstruct the path of investigation. Diseases, though individualized by names, are not simple entities, having peculiar and well-defined characteristics by which they can be severally identified. Let any one, entering the wards of an hospital and noting down the leading symptoms presented by the various cases, compare them with the nosological definitions of Cullen or any other writer, and he will find it very difficult to assign to the great majority of them either "a local habitation or a name." A few will be found wanting in some one or more of the symptoms essential to their admission into any one of the apparently well-defined, yet really commingling classes; while in much the larger proportion the symptoms will be found so varied and, seemingly, so opposite, that the diseases which present them might with equal propriety be referred to this or to that order, genus, or species. I speak from experience; for the exercise is one in which I often employed myself in the days of my pupilage, and the results were such as to convince me of the exceeding difficulty to be met with in forming a comprehensive, accurate, and (so to speak) natural nosology.

But much, if not all of this difficulty, it may be said, arises from confounding the essential and accidental, the constant and occasional, symptoms of diseases; and this objection may, to a certain extent, be valid: yet it is nevertheless true that diseases are, in general, much too complicated for even their essential characteristics to be comprehensively, and at the same time strictly and exclusively, embraced by nosological definitions. Hence we can scarcely ever hope to see effected that precision of arrangement spoken of by Sydenham, who says: "Primo expedit ut morbi omnes ad definitas ac certas species revocentur, eadem prorsus diligentia ac *akribeia*, qua id factum videmus a botanicis scriptoribus in suis phytologiis."

If we attempt to classify diseases according to the tissues which they severally affect, we shall soon find ourselves placed in positions of no small embarrassment. We shall find that diseases which affect in their origin one tissue exclusively, invade, during their progress, another, and by their ravages upon this latter proceed often to a fatal termination. Diseased heart, liver, or kidney, terminating in dropsy; dropsy terminating by gastric inflammation; asthma, phthisis, aneurism of the aorta, &c., giving rise to bronchitis; hypertrophy of the heart occasioning apoplexy—these are a few of the many familiar examples that might be adduced in illustration of this important truth. To which tissue then is a disease to be attributed?—to that primarily implicated? or to that, the affection of which is the immediate cause of death? It may indeed be alleged, that, in these cases, a second and distinct disease is superadded to the original one: but by this mode of regarding the question, practical utility, the grand and leading object of nosological classification, would be very little promoted; for morbid states and lesions, which in nature are generally found, if not co-existent, at least consequent and dependent one upon another, would thus be violently disjointed, and marshalled into widely distant classes. Thus diseases would be described in their purely simple state, a state in which they seldom occur, and cannot long exist; while those complications, in the detection and proper management of which medical sagacity and skill are chiefly employed, would be in a great measure overlooked or neglected.

But with all these objections to a nosology based upon the tissues, and framed according to our knowledge of the morbid changes which they undergo, I am yet firmly convinced that upon no other groundwork, and with no other materials, can any thing approaching to a natural classification of diseases ever be erected.

There are many, however, (and among them some of no slight pretensions) who ridicule nosology in the abstract;—who regard it as, at best, an ingenious exercise of the fancy, requiring in him who labours at it little more than a knowledge of the names of diseases;—who ask triumphantly wherein its practical utility consists; and while they cannot themselves answer the question, are too proud and self-conceited to listen attentively to the answer of another. With such men the justly-earned fame of Cullen is a jest, and the learning of the older lights of our profession is little better than obsolete nonsense. Well is it for the reputation and usefulness of our art, that such opinions were not prevalent in former times; for then the records of medical science would have been as meagre as indolence could wish them, and their language as vulgar as ignorance itself could desire.

If no other advantage arose from our attempts to classify diseases, there would yet result this important one,—that we should thereby be taught the existing limits of our knowledge; for the degree of success attendant upon such attempts appears to me at least the most certain test of our actual proficiency. Before diseases can be arranged in any thing like a natural order, it is necessary not only that their own natures be correctly and intimately known, but also that their natural affinities be thoroughly understood. It is idle therefore to deny, that he who frames a good system of nosology must of necessity be practically and minutely acquainted with disease.

Nothing, however, could be more absurd, than to make an acquaintance with any one or more nosological systems a decisive criterion by which to judge of the qualifications of candidates for professional honours or practice. Any man may get by rote what he does not comprehend, while it is perfectly possible for a practitioner to understand thoroughly, and treat judiciously, diseases of which he does not even know the names. One cannot, indeed, be long and attentively conversant with disease, without framing in his own mind a nosological system of some kind or other; imperfect, it may be, and ill-arranged, but yet sufficiently clear and complete to facilitate his diagnosis and aid him in his practice. Such a system must, from the very manner of its formation, be constructed upon the analogies and natural affinities of diseases, without due attention to which no truly accurate and useful system of nosology can possibly be framed.

I have dwelt at some length upon the question above discussed, from a belief that the true end and scope of nosological classification are not generally understood; and that its importance, therefore, is not duly appreciated. I have pointed out a few of the difficulties incidental to its formation—difficulties which, for the most part, arise from our imperfect knowledge of the subjects which it embraces, but which must diminish, and finally even disappear, in proportion as the light of medical science increases. I have expressed my conviction that a knowledge of the tissues, in their normal and morbid states, must form *the best basis on which to ground a correct system of nosology*; and I have endeavoured to show, that the success attendant upon any attempt to classify diseases must form a sure test of the professional knowledge of its author, and (supposing him well informed) of the scientific advancement also of the age in which he lives. As then nothing is better calculated to promote knowledge than to demonstrate to men wherein, and to what extent, they are ignorant, it is very much to be desired that some one, fitted for the task by talent, observation, experience, and learning, will undertake the formation of a nosological system, founded upon the discoveries, and executed according to the advanced knowledge, of modern times.

In making out the foregoing tables, I have arranged the diseases simply according to their localities,—a plan which made it necessary to assign to *fevers* a special and distinct order, inasmuch as they are essentially diseases of the general system, although aggravated often by local complications. In this order I have comprehended two diseases not hitherto looked upon as fevers, but which, from their general nature, I have long regarded as such. This departure from ordinary custom, as it is made without pretension, will, I hope, be passed by without censure.

In treating of the diseases included in the tables, I shall follow the same order adopted in them; and while, for obvious reasons, my Report shall treat chiefly of the in-patient cases, notice will be occasionally taken of such interesting facts and observations as stand recorded in the out-patients' book.

Every disease however common, and every case of disease however unattended with unusual symptoms, must possess an interest for him who really loves his profession; who studies medicine for its own sake, and for the practical good which flows from it, rather than from the selfish wish to acquire reputation. For while it cannot be denied, that the more minutely and accurately we are acquainted with the nature and symptoms of a disease, the more rationally and efficiently are we likely to treat it; we may, at the same time, without fear of contradiction assert, that even in the most familiar maladies, there are still points, both theoretical and practical, which not only admit of, but require, further investigation. It behoves therefore the diligent cultivator of our science to observe closely, and report faithfully, *all* the morbid phenomena that present themselves; and not to limit his notice to those only that are calculated to surprise by their novelty, or to ensure general attention by their great and manifest importance.

But though I thus insist on the propriety of not overlooking and neglecting diseases and phenomena of disease, merely because they are of frequent and ordinary occurrence, I cannot help thinking that much harm has resulted from the too prevalent practice of diffusely describing diseases by dwelling at length on particulars which, being neither essential nor constant, had much better be omitted. This error is most conspicuous in the recorded histories of "cases," in which are usually found related (sometimes with great precision and an air of no slight importance) facts and circumstances which neither have any real connexion with the cases, nor reflect any light upon them.

Anxious to avoid *both* the errors above specified, I dictated on the admission of each patient, whether in or out, an account of his *then actual state*; for the most part merely mentioning the symptoms, in the order of their prominence, with scarcely any connecting words, and altogether without interspersed thoughts or observations. Thus the record of each case bore a strong resemblance to a nosological definition, from which, however, it differed materially in that it presented, generally, a greater or less complication of the symptoms of more than one nosological disease. To such a statement of the existing malady, was added a brief mention of its duration and supposed cause, as well as a concise history of its progress up to the period of admission. A similar method was pursued in noting its subsequent course; so that the whole together constituted, as it were, but a skeleton of the case, all those parts which are usually added to give form, and roundness, and an attractive appearance, but which seem to me partially to conceal the fundamental and essential nature of a disease, being intentionally omitted. This much I have thought it necessary to say in justification of my own proceeding, and with that view solely; having no wish to prescribe a plan of conduct for others engaged in similar pursuits.

### CHAPTER I.—OF FEVERS.

It very seldom happens that a practitioner finds any difficulty in pronouncing on a case of fever; and yet, up to the present day, the greatest lights of our own profession have been unable to determine wherein *the essence of fever consists*. Nor is this all, for even its seat is still undecided, pathological anatomy, from which one might have expected a solution of the question, having notably increased the grounds of dispute. It is not my intention to enter into an examination of the numerous theories of fever which have at different times been promulgated, and which, while they have received the assent and admiration of some, have been denied, combated, and even ridiculed by others. The subject is, indeed, one of importance; but it is better suited to a systematic treatise, than to a clinical report.

A mere detail however of isolated facts, unconnected by any chain of reasoning, and from which no conclusions are drawn, is very little calculated to add either to the extent, or the accuracy of our knowledge. While, therefore, I regard as foreign to my design, a critical enquiry into the opinions of authors respecting the diseases treated of in these pages, I feel that I may, without impropriety, not only state in general terms what my own views are, but also support them, when necessary, with appropriate arguments. In doing so, I must occasionally come into collision with the opinions and statements of others; in which case I shall consider myself justified in commenting on them so far as may be necessary to remove any impediment they may offer to the admission of my own views.

With respect to fever, the following propositions may, I think, be maintained and proved.

1. Fever is a general disease, affecting every organ and every tissue in the body.

2. Its acknowledged primary symptoms declare a depressed and actually enfeebled state of the whole nervous system.

3. But previously to the appearance of these symptoms, *the organic system of nerves, and the nutritive functions over which it presides,* have almost always been implicated, causing a deterioration of health, both felt by the patient, and visible to the eye of an attentive observer, although unattended with very prominent symptoms. Thus an affection of the organic system of nerves, and an almost simultaneously deranged state of the whole of the nutritive functions, constitute the first link in the chain of febrile lesions, and may, indeed, be considered as *the essence* of the disease; for as it is the first, so is it the only constant lesion in fever, through every stage of which it exists continuously.

The only exception to the statement, with which this proposition commences, is to be found in those cases of fever which originate in the action of powerful poisons, or in some sudden and violent impression made upon the nervous system generally; and this exception, as will be afterwards proved, constitutes, in reality, no objection to our doctrine respecting the *essence* of fever.

4. The local lesions which have, severally, been regarded as the cause, or as constituting in fact the essence of fever, may, and often do exist without the presence of fever: for the pyrexial excitement which they occasion is merely one, and that too a non-essential, element of fever. Individually they are not always present, and collectively they are sometimes absent. From all of which considerations, it follows, that they must, in general, be looked upon as either accidental or consequential. I say *in general,* for it sometimes happens that the local lesions, having existed for a considerable time, impair the energy of the nutritive functions, and become thus virtually a cause of fever.

Let us examine these propositions, and compare the doctrines which they express with universally admitted facts.

Prop. 1. The general, or as it has inaptly been named, particularly by the French writers, the *essential* nature of fever, is sufficiently proved by an enumeration of its symptoms. These declare a more or less disordered state of all the functions—of digestion, respiration, secretion, absorption, and the circulation; of the cerebral, nervous, and muscular functions. Some are affected at an earlier period, and to a greater degree, than others; but no case of fever passes through its whole course without presenting a greater or less derangement of them all. Most of them have their activity impaired; but the circulation, after what is considered the primary stage of fever has passed over, is, on the contrary, unnaturally rapid; and during the whole progress of fever absorption would appear to go on with augmented activity. This, however, may be only apparent; for nutrition being nearly at a stand, the action of the absorbent system must consequently be more visible in its effects.

Now as all the functions of the body are thus manifestly disordered, we are necessarily led to conclude, that the organs by which those functions are performed are in some way or other altered from their natural state; although such alteration may not be appreciable to our senses. And as in the composition of these several organs (all severely though not equally affected) different tissues predominate, there can be no reason to believe that any tissue is exempt from the morbid influence. Fever must, therefore, be regarded as a disease of the whole system.

Prop. 2. The accession of fever is marked by loss of appetite, nausea, chilliness, paleness of the surface, lassitude, indisposition to, and incapacity for, much mental exertion. These symptoms may however be produced by causes which, transient in their operation, do not give rise to actual fever, although they occasion a state of the system similar to that which exists in one stage of fever. Among the causes here alluded to, may be mentioned prolonged vigilance, abstinence, simple fatigue, a debauch, intestinal irritation, &c., all capable, individually, of producing actual fever, if frequently repeated; although a single operation of them goes no further than to occasion a depressed and disordered state of the nervous system. But in fever the nervous system is not only depressed, it is actually enfeebled, and cannot, therefore, be restored to a healthy state without the intervention of those other changes which by their occurrence constitute the subsequent stages of fever.

Prop. 3. The symptoms detailed in the foregoing paragraph denote an enfeebled state of the *whole* nervous system—of that part which presides over the functions of relation, as well as of that appropriated solely to the maintenance of organic life. But antecedently to the appearance of such symptoms there has existed, for a longer or shorter time, a deranged condition of the organic system of nerves, as well as deranged action of all the organs employed in the several processes of nutrition, from the digestion of the food to its conversion, from the blood, into the solid components of the body. The period during which this deranged action may exist without affecting the brain and nervous system generally, is uncertain; but it is not, most probably, very long, for as the blood itself is speedily rendered impure, it must soon cease to afford a healthy stimulus to the several organs. Thus we can readily account for those symptoms which attend upon what is usually considered as the first stage of fever, all of which are attributable to a state of positive general debility, induced by an almost complete arrest of the several processes of nutrition.

The foregoing truths, while they afford a satisfactory explanation of what is called the *latent period* of fevers, receives, at the same time, no inconsiderable support from it. We cannot suppose that the poisons which produce certain kinds of fever, and which remain each a determinate period in the system, before giving rise to what are considered the initiatory symptoms of fever, are in the mean time totally inactive. The indescribable state of *malaise*, in which patients find themselves during the latent period, renders such a supposition impossible. What then can be more rational than the conclusion, that the poisons, while they thus exist in the body, are employed in overcoming its organic powers, and that when they have effected this (which they do in a longer or shorter period, according to their several energies) their own operation begins to be manifested. Some are sufficiently strong to produce their full operation almost instantaneously; but even then their ultimate effects are the same, although their immediate mode of action is somewhat different. Thus, in these cases, the first distinguishable impression may be on the nerves of sensation; but those of the organic system are speedily implicated, and the whole organic functions consequently impaired. Were it otherwise, the effects of the poisons would be at once partial and transitory.

From what has been said, it may be easily understood how every cause capable of weakening the organic powers of the system, may, by its continued action, induce gradually the same degree of imperfect nutrition and consequent general debility, which the more potent poisons, as it were, instantly give rise to.

Prop. 4. The arguments adduced by me in confirmation of the foregoing opinions, are derived from the history of fever—from the causes which produce it—the manner in which they operate—the symptoms which they occasion — and the order in which those symptoms present themselves. Evidence of this kind is much more to be trusted than mere pathological appearances, which declare the results of the several morbid processes, without affording us much insight into their order of succession, or their dependence one upon another. I am not, I hope, insensible to the advantages that are to be derived from a knowledge of morbid anatomy; but it is certain, that an implicit confidence in its evidence often leads to erroneous opinions. The disease of which we here treat affords, more perhaps than any other, ample proof of the truth of this remark.

That in certain types of fever, the mucous membrane lining the stomach and bowels is often extensively diseased cannot be doubted; nor is it less unquestionable that in other types (particularly in that denominated typhus) the brain is very generally found more or less altered in its structure: but the doctrine that, in either case, the local lesion is the cause, or constitutes the essence of the disease, is totally irreconcileable with the history of the symptoms, as well as with the facts that such lesions often exist without fever; while fever, again, frequently runs its course, even to a fatal termination, without presenting, after death, any visible traces of local disease.

After what has been said of the essential nature of fever, we need not, I think, have any difficulty in accounting satisfactorily for the frequent occurrence in it of intestinal ulcerations. These are a necessary consequence of imperfect nutrition, by whatever cause produced; and are, therefore, commonly met with not in fever only, but in phthisis (in which, according to the accurate Louis, they exist in five-sixths of the cases) as well as in *tabes mesenterica*, and, indeed, more or less frequently, in most chronic diseases. In the animals fed on bread and water by Majendie, ulcerations of the conjunctival mucous membrane always presented themselves. The process of ulceration consists essentially in a diminution or complete arrest of the organic or formative action of the capillary vessels; while the absorbent action goes on in the natural way. It takes place only in debilitated subjects, or, at least, in debilitated parts; and (if we except the skin) is most common in the mucous membranes, the lax and delicate vessels of which easily admit of congestion, and thus, losing their tone, have their formative action speedily impaired. The comparative frequency with which the digestive mucous membranes are thus affected may, in some degree at least, be accounted for by the slowness of the circulation through the portal system, on precisely the same principle as that by which we attribute ulcerations on the lower extremities to a varicose state of their veins.

Next after the mucous membranes, the tissue that most readily becomes ulcerated is that of cartilage; for its powers of life being naturally low, any further reduction, by the occurrence of inflammation or otherwise, speedily destroys the formative action of its vessels. Hence the frequency with which the cartilages are ulcerated in scrofula—a disease of general debility and great want of tone, in both of which the cartilages must of necessity participate.

The great importance attached to the intestinal ulcerations of fever, of which disease they are by so many regarded as the cause, have induced me to dwell thus at length, not only upon them specially, but also upon the ulcerative process generally, with a view to prove that as *it* owes chiefly its production either to general or local debility, inducing a diminution or arrest of the formative action; so they ought, in most cases, to be regarded as the consequence of fever—a disease the very essence of which consists, as has been shown, in a weakened state and disordered action of the whole nutritive capillary system.

But it is nevertheless certain that intestinal ulcerations not only augment the fever, by impeding still further the process of general nutrition; but that they may, if brought into existence by any other debilitating cause, actually induce fever. This will not surprise us, when we consider how much ulcerations so seated must impede nutrition by interfering seriously with its first, namely, the digestive process. Thus are we enabled to account for the fever which frequently terminates phthisis, as also for that which often forms the closing scene in malignant and other chronic diseases. It is not meant that, in the above-mentioned instances, the ulcerations cause fever merely by their direct influence on digestion; they act also by exciting a sympathetic affection of the whole organic system of nerves.*

### SIMPLE CONTINUED FEVER.

Of this form of fever only a single case was admitted. The patient, an unmarried female, æt. 22 years, could assign no cause for her illness, which had existed three weeks. Symptoms on admission (Sept. 9), head-ache, pain in epigastrium and in left iliac region, prostration of strength, countenance anxious, eyes slightly suffused, appetite impaired, tongue covered with a thick brown fur, bowels costive, pulse 100 feeble, cat. regular.

Previous to admission, she had been much troubled with nausea and vomiting, the former of which often recurred during her residence in the hospital, and proved very troublesome. Soon after the disease commenced, she had been bled from the arm without benefit.

---

* That these notions respecting the nature of fever have not been hastily formed by me, may be proved by reference to an article on the subject, which published in the *Medical Gazette*, more than five years since. *Vide Med. Gazette* for June, 1832; also *Lond. Med. and Surg. Journal* for (I think) Nov. in the same year.

The treatment consisted of mild aperients, leeches behind the ears, blisters to the epigastrium and nucha, emetics, and, on the decline of the disease, quinine. The emetics were given at a more advanced stage of the disease than it is usual to exhibit them (namely, at the end of the third and fifth weeks after admission), and proved very serviceable, cleaning the tongue, and relieving both the nausea and head-ache. The epigastric pain and tenderness prevented me from prescribing them at the outset. Their good effects were scarcely visible until the second day after they had been taken.

On the 28th of October, the patient was in a fit state to be discharged; but as she was still weak, and I wished to watch her convalescence, she was made an out-patient; and, going into the country, soon regained both flesh and strength.

*Remarks.* This was a case of that form of disease which some have denominated "walking fever;" for during the whole time that the patient remained in hospital, she was not confined to bed a single day. It was rendered tedious by the complication of head and gastro-enteric symptoms which, for three weeks, regularly alternated, the one set always declining as the other increased. This I have remarked in many other cases of fever.

The form of fever here treated of readily yields (as I have often experienced) to the liberal use of bark or quinine, when there is not much gastric or intestinal derangement; but in the present case, neither of these remedies could be employed until a very late period of the disease.

### Typhoid Fever.

Of this form of fever only the following case was admitted.

Daniel Linwood, æt. 18, admitted April 22nd. *Symptoms.*—Low delirium; countenance pale; eyes suffused; epigastrium tense, and, as well as whole of abdomen, very tender on pressure; tongue dry, brown in centre; pulse scarcely perceptible; urine passed involuntarily; extremities cold.

Five weeks previously to admission had been discharged the hospital, where he had been under the care of my colleague, Dr. Chisholm, for an affection of the chest. His health continued impaired until the 17th of April, when he was seized with head-ache, prostration of strength, aversion to food, &c. He died on the day after admission, at 8 o'clock P. M.

*Inspection 39 hours after death.* Meningeal arteries filled with black blood; arachnoid opake and thickened; with subjacent patches of semifluid coagulable lymph; substance of brain extremely vascular; about two drachms of serum in left lateral ventricle, and upwards of two ounces in base of skull.

Bronchial ramifications throughout the whole of both lungs much dilated, and containing muco-purulent matter. A few granular tubercles visible near the surface of upper lobe of right lung.

Mucous membrane lining stomach of a mottled appearance, thickened, softened, and easily separated from middle coat.

*Remarks.*—We have here a case of typhoid fever, succeeding to chronic bronchitis, and carrying off the patient in six days after its access. The morbid appearances presented by the brain and stomach were unquestionably produced within this period, most probably within a few days of death, while those of the lungs must have had a much earlier origin. The patient, at the time of his admission, was evidently moribund; and about two tea-spoonsful of fluid were all that he could be got to swallow, during the few hours that he survived.

### Chlorotic Fever.

Chlorosis has been regarded, by different authors, as a species of *cachexia*,—as one of the many forms of *hysteria*,—as a result of *amenorrhœa*,—as, simply, a consequence of *anæmia*. The first of these views is that generally entertained by the older authors (in whose writings therefore, the disease must be sought for under the head of *cachexia*). The second is advocated by Sydenham. The third by Cullen. And the last by many of the Continental writers of the present day. But chlorosis may be said rather to comprehend within itself all the morbid states so named, than to be allied to, or result from, any one of them. I have ranked it among the fevers for the following reasons:

1st. It is a general disease, affecting every organ, and every tissue of the body.

2nd. It consists essentially in a deranged and actually enfeebled state of the organic system of nerves, and of the nutritive functions over which it presides.

The truths contained in the above propositions, are rendered, I think, manifest by a full and accurate consideration of the symptoms of the disease. But before proceeding to enumerate these, it may be well to state that I reject as altogether fanciful the division of chlorosis into two species, the *entonic* and the *atonic*. The former I believe, has no existence in nature.

The first or *latent* stage of chlorotic, like that of every other fever, is marked by no very prominent symptoms. Languor, depression of spirits, impaired heat of surface, broken sleep, disordered digestion—these exist as objects of feeling, for a considerable time before they or their effects become manifest to observation.

When the disease is fully developed, the following state of the system presents itself:

Skin dry, of a pale, yellowish, or greenish tinge; countenance heavy; eyes dull; under eye-lids tumid, particularly in the morning, often livid; lips exsanguious; great prostration of muscular power, giving rise to pain in left side, in neck, back, and extensor muscles of lower extremities; depression of spirits; chills, especially towards evening, and followed by exacerbations of fever; drowsiness; pain in occiput, often in left temple; short, hurried, irregular respiration; palpitation of heart; tendency to swooning; diminished, often depraved, appetite; acidity of stomach, nausea, and sometimes vomiting; flatulency, with distention of abdomen, and costiveness of bowels; a suppressed or disordered state of the menstrual secretion; œdema of ancles; coldness of feet; weakness, and febrile quickness of pulse.

In a still more advanced stage of the disease, the patient is querulous and fretful; the skin becomes rough, of a dirty, dull, leaden hue; respiration is not only quick and hurried, but laborious also; there is cough with scanty mucous expectoration, sometimes slightly streaked with blood; the heart beats more feebly, quickly, and irregularly; the abdomen is permanently enlarged, and fluctuates; and the bowels are irregularly loose.

All these symptoms may exist, and yet the patient, by proper treatment, recover; but the following *I have always observed* succeeded by a fatal issue —a swelling of the whole of one lower extremity (the left is that most frequently so affected), and inability to sleep except in a semi-erect posture.

From the symptoms above detailed we perceive that, in chlorosis, the muscular, respiratory, nervous, sanguineous, secreting, excreting, and absorbent systems are all extensively and seriously implicated. In what *other* class of diseases than the *fevers*, is this universality of sympathy observed?

The form of fever, to which the chlorotic bears most analogy, is the remittent fever of children; which, indeed, has been named by some authors *chlorosis infantium* (vide Suavages), but this latter is now almost universally ranked among the fevers.

In chlorosis the absorbent system is less active, and the sensorial functions less affected, than in most of the other forms of fever: but these, if real objections to my views, apply in an equal degree to remittent fever, as the latter does to gastric fever also.

My doctrine of the febrile nature of chlorosis, derived as it has been from attentive observation of, and thoughtful reflection upon, the symptoms, receives no slender confirmation from the fact, that Sydenham, in treating of the disease, designates it by the synonyme "febris alba."—(see his letter to Dr. Cole, in *Oper. Univers*, p. 354, Lipsiæ, 1827.) In his *Processus Integri*, he gives as one of the symptoms, "pulsus febrilis." Hoffmann also describes the disease as accompanied, in its advanced stage, by a slow fever—"increscente malo, lenta se associat febris, noctu maxime affligens, &c." (De Chlorosis Indole, &c. in Oper. Omn. Supplemento, T. vi. p. 391. Genevæ, 1753). Platerus, in treating of fevers, describes a well-marked case of chlorosis under the head of, "Febris Lenta, Cachexia, Cordis et Arteriarum Pulsatio." (*Observ. Libri Tres. p.* 355, *Basileæ*, 1641.)

Some authors describe chlorosis as affecting occasionally the male subject; but I have myself never met with such a case. How then, if the disease be not in some way or other dependent upon the genital organs, is this fact to be accounted for? The general constitution, education, and habits of life, peculiar to females, may be stated in answer.

Dr. Cullen evidently regarded chlorosis as the product of *retained* menses, —a notion to which there are these two strong objections:—first, the disease is not confined to the period of puberty, but is very commonly met with in patients from 16 to 21 years of age, with whom the catamenia had for some time flowed regularly: and, secondly, cases not unfrequently occur in which the menstrual discharge continues to appear at proper intervals, although in diminished quantity, and of an unhealthy quality. This I have observed to happen in some very severe cases.

The true light in which to regard the retention, suppression, or altered state of the menses in chlorotic fever, is to look upon it as the result of the fever, and of the co-existent general debility. No one now regards suppression of the catamenia in phthisis as the *cause* of that disease; or attempts, as some of the older physicians advised, to cure the affection of the lungs by endeavouring to restore the uterine secretion. Almost all the remedies which were, and, by a few, still are, considered to exercise a peculiar and direct influence upon the uterus, act upon it solely through the medium of the general system.

Roche also (Dict. de Med. et Chir. Prat.) considers an enfeebled and inactive state (*langueur*) of the genital organs as the cause of chlorosis; but, in my opinion, a disordered state of the assimilative organs might, with much more reason, be assumed as its origin. These, as Hoffman well observes, are in a state of atony, giving rise to sluggish circulation, diminished secretion and excretion, impure chyle, depraved blood, impaired and disordered nutrition. (Op. Citat. p. 392). Only one link, but that the primary one, viz. an enfeebled state of the organic system of nerves, is here wanting to complete the chain of actual morbid processes.

Van Swieten attributes chlorosis to " a defect of the action of the solids on the fluids," and to an " imperfect elaboration of the chyle into blood." Now these morbid states certainly do exist in chlorosis; but then they constitute only a *part* of the general pathological condition, and are, moreover, *consequences* of the pre-existent cause mentioned in the foregoing paragraph.

Hysteria, as well as chlorotic fever, depends upon debility of the organic powers: but in the former the debility is minor in degree, and does not lead to that general and aggravated disorder of the nutritive functions in which chlorotic fever essentially consists. We are thus enabled to account for so many symptoms being common to both, as also for the transient nature, and greater momentary severity, of those presented by hysteria. In chlorotic fever, the frame is not vigorous enough to originate a very high degree of action.

Among the symptoms common to hysteria and chlorotic fever, there is one, which, for its practical importance, deserves particular notice. It is a tender state of the spine, produced unquestionably by a sympathetic affection of the nerves of sensation. Depraved secretions in the digestive canal, together with flatulent distention of the intestinal coats, are well calculated to irritate the extremities of the sensitive nerves; and *it has been long known*, that irritation of the extremity of a nerve often produces a morbid state (I purposely avoid the term congestion) of its origin. It is possible, also, that there may be a direct communication of diseased influence from the sympathetic to the spinal sensitive nerves.

In some works published within the last few years, the spinal tenderness here spoken of is regarded as the result of original spinal irritation; and the disordered digestion, hurried breathing, palpitation of heart, &c. are set down as consequences also of the same. But this doctrine appears to me as irrational in theory, as it is seriously pernicious in practice.

There can be no greater error than the belief, that, because the local pains of hysteria are temporarily relieved by leeching, leeches are, therefore, either necessary or beneficial. I would not be understood as altogether condemning their use in this disease; but I very rarely indeed find it necessary to prescribe them in it, and *I have seen many bad consequences result from their repeated application to hysterical patients.* Among these I would mention the production of a whole host of anomalous symptoms; of a peculiar species of paralysis (the hysterical); also of such a state of the system as gives rise to a loud demand for further depletion, even when the body to the eye appears almost exsanguious.

Another evil resulting from the doctrine of "spinal irritation" is, that exercise is usually prohibited; and the patients condemned, sometimes for months together, to a sofa; when a ride on horseback daily, with the administration of suitable general remedies, would speedily remove all traces of local, as well as constitutional, disease.

The efficacy of exercise in preserving the health of females predisposed to chlorotic fever is well set forth by Van Swieten; who says, that when the patients have taken steel and purgative medicines for three or four weeks, "all the functions are quite restored; good wholesome blood begins to appear; and soon after the menses flow spontaneously at regular periods, provided they make use of that strength, which they have recovered by the use of this remedy (steel), in the motion and exercise of their bodies: for when they are cured, if they should again indulge themselves in drinking large quantities of warm watery infusions, and in a constant sedentary way of living, they will most certainly relapse into their former disorder."—*Comment.* v. xiii. p. 295, Ed. 1776.

One of the most striking features in chlorotic fever, is the unnatural tinge of the skin, which is owing not merely to the changes undergone by the blood (deprived as it is of a large portion of its *albumen* and red particles), but also to depraved nutrition from deficient energy of the nutritive capillaries.

Intermediate between chlorotic fever and simple anæmia, is a form of disease which, from the pure whiteness of the skin, has been denominated *leukosis*. In it the catamenia are seldom quite repressed, sometimes at the commencement they flow very profusely; the appetite is less impaired; nutrition is more healthily performed; and the febrile symptoms are therefore less distinctly marked. It is, as might be expected, more easily cured than chlorotic fever.

The prognosis of this disease is often rendered very difficult by its apparent complication with tubercles in the lungs: and although a careful consideration of the whole of the general symptoms, enables us, in most cases, to form an accurate judgment, yet, but for the aids which percussion and auscultation afford, we should now and then find it impossible to do so. Even with such aids, discriminating physicians do sometimes fall into temporary mistakes. I have seen chlorotic young females who, after presenting difficult breathing, hæmoptysis, discharge of blood by stool, extreme emaciation, night-sweats, &c., have died of effusion into the pleura and pericardium, and in whose lungs not a trace of tubercular matter could be discovered after death. Who will say that he might not be mistaken in such cases?

By a reference to the tables it will be seen, that among the in-patients, there were eleven cases of chlorotic fever, and exactly the same number among the out-patients. The average age of the former was 22 years; of the latter 18 years. Among the former was one patient (a widow) æt. 44 years, which will in some measure account for their average age being so much greater. The former were, on an average 51 days under treatment: the latter 68 days. Both these periods may appear long; but most of the cases were very severe, and had had a protracted existence previously to their admission. To the good fare of the hospital must be attributed in some degree the shorter continuance under treatment of the in-patients.

The catamenia were present, though scanty and deranged, in *ten* cases; totally suppressed in *eight*, and retained in *four*. They were present in some patients in whom the disease existed in a very severe form.

The remedies employed were the same in almost all the cases, and were scarcely ever changed throughout the whole period of treatment. Some preparation of iron, generally the tinct. ferri muriat., or an electuary of the *newly-prepared* carbonate, with a purgative twice a week at least, formed the leading remedies; while for the pain of head an emetic was sometimes prescribed with great benefit; or if that failed to give permanent relief, a small blister was applied to the temple; and, the cuticle being removed, half a grain of powdered morphine was dusted over the raw surface. Nothing could exceed the good effects of this measure, which was found equally efficacious against the troublesome palpitations of heart that prevail in chlorotic fever. In a few patients the morphine, thus applied, produced nausea and retching, an occasional inconvenience attendant also upon its internal use. I am not aware that any preceding writer has mentioned this curious fact. There is one family of my acquaintance, every member of which is attacked with retching after taking the smallest dose of morphia; although laudanum is borne without inconvenience. The head would appear to suffer first, and through it, the stomach.

I have hinted above at the propriety of combining frequent purgation with the use of steel in this disease. The same rule holds good in every instance in which any of the preparations of steel is administered. Without attention to it, feverish heat, headache, &c. are always excited, and the patient is rendered weaker, rather than strengthened. So strongly am I convinced of this truth, that I always regard frequent moderate purgation as an essential part of the treatment by steel. Frequently I give the purgative and steel in combination, as the ferri sulph. with the magnes. sulph. or the tinct. ferri muriat. with the tinct. aloes. The following draught is one which I often prescribe in chlorotic fever.

℞. Tinct. ferri muriat. ʒss.

—— hellebori nigri, ʒj.

—— aloes, ʒss. ad ʒj.

Inf. quassiæ, ℥x.

M. ft. haustus ter de die sumendus.

Even when aperients are thus combined with the steel, the separate exhibition of a purgative is often called for. Should however the stools at any time contain either blood or masses of gelatinous looking matter, both the purgatives and the steel ought to be immediately omitted for a time, and reliance placed solely on an unstimulating nutritious diet with exercise proportioned to the patient's strength.

Having made these general observations, which, if lengthy and numerous, are yet not unsuited to the importance of the disease; I shall now proceed to detail briefly a few of the cases. Where nothing to the contrary is said, it is to be understood that the subject of the case is an in-patient. This remark holds good with regard also to every other part of the Report.

*Case* 1.—S. M. æt. 21, admitted April 22nd.—*Symptoms.*—Countenance of a pale yellowish cast; eyes dull; conjunctivæ without bloodvessels; lips exsanguineous; respiration short and hurried; cough, with mucous, occasionally bloody, expectoration; palpitation of heart; pains in shoulders and in both sides; profuse night sweats; appetite much impaired; tongue slightly furred; bowels costive; pulse 120 feeble; cat. regular, but scanty and pale. Bronchophony under both clavicles, but percussion clear.

Illness had existed two years.

*Treatment.*—Mist. ferri comp. ℥j. Tinct. digitalis, gtt. x.—ft. haustus ter de die sumendus.

Diet generous.

May 2. Sumantur c̄ sing. haust. pil. ferri comp. gr. v.

25th. Health and strength have progressively improved. Patient has gathered flesh, and her countenance has resumed its natural complexion. Night perspirations gone. Pulse 74. Natural functions all healthily performed.

Two days after this report, she was made an out-patient, and was soon completely restored to health.

*Remarks.* This was one of those cases in which we find it very difficult to decide upon the existence or-non-existence of tubercles. The hæmoptysis and night sweats would lead to the former inference; the absence of dulness on percussing the clavicles to the latter. The bronchophony unaccompanied by this last symptom could be readily accounted for, by the emaciated state of the patient. It appeared, however, more distinct under the left clavicle; but the difference was so slight, that I thought it might be imaginary. I have therefore not noticed it in the report.

The digitalis was continued throughout the whole of the treatment. It is a very mistaken notion to suppose that this medicine, in moderate doses, has a debilitating influence.

With the exception of occasional mild purgatives, no other remedies than those mentioned were employed. Their good effects were very striking. In a few days, the cough and night sweats ceased; the breathing soon became more regular; the palpitations diminished, and gradually altogether subsided; the pulse sank to 74, making a difference of 46 beats in the minute; the appetite became natural; and the patient, in little more than a month from her admission, went out of the hospital with plump cheeks and a florid complexion.

*Case 2.*—J. E. æt. 17, admitted May 27th. *Symptoms.*—Countenance pale and sallow; frequent dry cough; hurried breathing; palpitation of heart; pain in left temple; also in left side, and back; spine tender, particularly over two lowermost dorsal vertebræ; ancles tumid; feet habitually cold; bowels costive; tongue furred; pulse 100, feeble; cat. regular, but secretion scanty and pale.

Attributes her illness to cold caught twelve months since. Has been bled from the arm three times, and cupped along the spine ten times, besides being repeatedly blistered in the same region.

*Treatment.* Tinct. ferri mur. ʒss. ex infuso quassiæ, ℥j. ter de die.—Pil. colocynth. comp. gr. v. singulis noctibus.

June 6th. Considerable improvement. Tenderness of spine diminished. Head-ache with absence of fulness and obstruction (as it were) in epigastrium; tongue furred; pulse sharp.

Medicines to be temporarily omitted—sumat vespere pulv. ipecac. ℈j.

On the following day, the head and epigastric symptoms had disappeared. The steel medicine and pills not however resumed till the 8th.

15th. Head again painful—*emp. lyttæ nuchæ.*

18th. Head still affected—*balneum pluviale singulis auroris.*

24th. Return of epigastric fulness, &c.—Vespere pulv. ipecac. ℈j.

From this date the patient's recovery progressed uninterruptedly, and she was discharged cured on the 8th of July—five weeks from the time of admission.

*Remarks.* We have here an instructive instance of the evil effects that result from treating the spinal tenderness of chlorotic fever as *an original and independent affection.* Who can doubt that so much depletion must have aggravated and kept up the symptoms? I believe, indeed, that the disease, as it existed on the patient's admission, was, in a great measure, the *creature of bad treatment.* The spine soon ceased to be tender *when nothing was done to it,* and when the patient's health was properly attended to.

The medicines prescribed on the patient's admission were continued until the period of her discharge, without alteration; and also without intermission, except for two days (from the 6th till the 8th of June) during which there existed derangement of stomach, with head-ache, and a tendency to febrile action, all of which were speedily relieved by the emetic. The head-ache returned, and a blister was applied, but without any benefit. The shower-bath was then used, and its good effects soon became obvious. It was continued daily during the remainder of the patient's stay in hospital.

A second emetic exhibited on the 24th June proved not less beneficial than the first. Emetics, indeed, constitute a most useful adjuvant in the treatment of chlorotic fever. They not only relieve gastric embarrassment (to Anglicise a very significant French term); but impart a salutary stimulus to the whole of the system. Respiration, circulation, digestion, and the nervous functions, are all beneficially influenced by them, when judiciously administered.

*Case* 3.—A. R. æt. 19, admitted June 3rd. To the ordinary symptoms of chlorotic fever there was added in this case strabismus. The right eye was the one affected; but although its axis of vision seemed very different from that of the left, the patient did not see double. She saw in fact only with the stronger eye; and yet *when it was closed,* objects were still discerned, although less clearly by the other. There was acute pain in the right temple.

The yellow tinge of the skin was deep, not unlike that which attends upon declining jaundice: the bowels were obstinately costive; and the catamenia had been present only twice in six months—the last time four months previously to admission.

Illness had existed nine months; strabismus four weeks.

*Treatment.*—Tinct. ferri mur. ♏ xxv. ex infuso quassiæ ℥j. ter de die. Pil. hyd. c̄ coloc. gr. v. sing. noctibus.

On the 16th June an emetic was given with marked alleviation of head-ache, increase of appetite, &c. It was repeated on the 14th with equal benefit.

June 17. A blister to back of neck—one drachm of the steel electuary (containing 10 grains of the newly-prepared carbonate) to be taken with each draught.

23rd. Pil. hyd. gr. v. sing. noctibus, vice pil. hyd. c̄ colocynthide.

On the 16th July, the following report was made.

General health greatly improved since admission; strabismus much less visible; pain of head quite gone, but complains occasionally of giddiness; bowels regular; cat. have not appeared.

Pains in loins and hips being at this time felt, twelve leeches were applied *circa anum*, and the hip-bath used on their removal. Cat. did not appear, but the patient's general health continued to improve.

July 23rd. Pil. hyd. gr. v. nocte maneque.

July 25th. Pil. hyd. gr. x. sing. noctibus et gr. v. sing. auroris.

On the 1st of August the patient was attacked with influenza, which reduced her considerably. The steel draught and electuary were discontinued, and saline diaphoretics administered in their stead.

On the 15th the steel electuary was resumed, and with it a tonic draught containing quinine. Under this treatment the patient quickly rallied from the effects of the influenza, and on the 26th, was made an out-patient, her cure being complete in every respect, except that the menstrual secretion had not yet established itself. This however it did soon afterwards.

*Remarks.* This case was characterized by an unusually torpid state of most of the secreting and excreting organs—of the liver, the intestines, and still more especially of the uterus. The emetics, by rousing the system, operated very beneficially; their effects however were transient, and accordingly I found it necessary to prescribe the blue-pill. But although it was taken for some weeks, and in considerable doses, such was the torpor of the system that the gums were not in the least affected. Yet the yellow hue of the skin diminished under its use, and the bowels were kept by it in a regular state. The strabismus also completely disappeared; but this may have been owing, in part, to the tonic influence of the steel, which, up to the attack of influenza, was given without intermission.

It is in cases like the present, that stimulating injections into the vagina are most likely to be useful; but I have seldom seen any good follow their employment; and in such a state of the general system, it appears to me incomprehensible how a mere local stimulant can produce any decided beneficial effect. The arrest of the catamenial secretion is *an effect of the general disease;* and while that disease continues, any action that we may be able to force the uterus into by stimulating injections must be both imperfect and very transitory.

*Case* 4.—F. R. æt. 24, admitted June 3rd. *Symptoms.*—Great general debility; skin dry, harsh, and of a brownish yellow tinge; eyes surrounded with a dark brown circle; hurried breathing; frequent palpitations, tenderness over two superior lumbar vertebræ; pain and numbness of both lower extremities; inability to walk even a few steps, although, in the horizontal position, the limbs are moved with freedom; skin covering legs traversed with numerous large blue veins, and marked anteriorly by large, slightly elevated, reddish brown patches, the remains of long-continued *erythema*

*nodosum*; adjoining periosteum thickened and uneven to the touch; pulse 84, feeble; tongue of a uniform bright red colour; gums soft and spongy; bowels regular; cat. absent for six months.

Illness commenced very suddenly about eight months since, when she was bled from the arm twice.

*Treatment.*—Pulv. alterativ. gr. v. sing. noctibus. Tinct. ferri mur. ℥iij. Tinct. hellebor. nigr. ℥ij. Dec. aloes c. ℥j. Inf. quassiæ ℥xss.—Ft. mist. ℥j. ter de die.

June 8th.—Omittatur pulv. alterativ. Hyd. submur. gr. xij. Pulv. opii gr. iv. Confect. q. s.—fiant pilulæ sex, sumt. j. sing. noct.

14th.—Slight irritation of bowels; faint effort at menstruation.—Omittantur pilulæ pro tempore.

15th.—Pain in region of duodenum.—Adhibeatur emp. lyttæ. Pil. Plummeri, gr. v. sing. noctibus.

17th.—Bowels free from all uneasiness.—Elect. ferri ʒj. ter de die.

20th.—Legs to be rubbed with the soap liniment, and bandaged with a flannel roller.

July 7th.—Electuary to be taken four times daily.

18th.—Pains still felt in tibiæ; periosteum thickened and uneven, although not nearly so much so as on admission.—Plummer's pill omitted; calomel and opium resumed.

From this date, the amendment was very rapid; and before the end of the month, the patient might have been discharged, but I was anxious first to see the catamenial secretion perfectly re-established. It appeared again on the 29th, but ceased on the second day after, the patient being attacked with acute catarrh, which threw her back more than five weeks. The steel mixture and electuary, and indeed all the medicine prescribed for the chlorotic fever, were omitted while the catarrhal symptoms were at the height.

On the 19th of September, the following note of the case was taken:

General health has very much improved during the last fortnight; and she is at present quite free from pain. Lower extremities have regained, to a considerable extent, their natural powers; which, for four months previously to her admission, were so much impaired that she could not walk across the floor. Bowels regular; tongue more natural in appearance, though still morbidly red; appetite good; sleep undisturbed and refreshing.

On the 23rd of September, the patient was discharged "benefitted;" for as the tongue was still morbidly red, and the catamenial secretion had not yet perfectly re-established itself, I did not deem myself justified in recording a cure. A few weeks afterwards I saw her at the hospital, when she appeared perfectly restored to health. She stated that she was then able to walk several miles without fatigue.

*Remarks.*—Each of the foregoing cases was characterized by some feature, or set of features, peculiar to itself—the first by hæmoptysis, night perspirations, and a strong general likeness to phthisis; the second, by an unusual degree of spinal tenderness; the third, by the existence of strabismus, together with an extremely torpid state of the general system. The present case was not less distinguished by an unusually morbid condition of the intestinal mucous membrane; by a spongy state of the gums; by the existence of *erythema nodosum*, and (probably as an effect of it) by thickening of the tibial periosteum. I suspect, however, that a strumous diathesis had something to do in the production of this last symptom, although there were no traces of glandular disease.

Having thus adverted to the nature of the disease, let us now consider briefly the treatment.

I make no comments on the venesection twice practised at the commencement of the patient's illness; for I know not how far it may have been necessary. I cannot, however, lose sight of the fact, that the attack for which she was bled was a very sudden one.

From the symptoms, as they existed on admission, I derived two leading indications—to impart tone and strength to the general system, and to improve the highly morbid condition of the intestinal mucous membranes. Each of these, indeed, might be said to include to a certain extent the other; but in practice it is often of great importance to separate *partially* lesions (and their representatives, symptoms), which in nature are indissolubly connected. Herein, indeed, consists one exercise of that practical tact, which bedside experience alone can give a man, and in which therefore the mere anatomical pathologist must needs be wofully deficient.

But to return to the treatment of the present case. Holding in view, but not over-estimating, the morbid condition of the intestinal mucous membrane, I prescribed for it the pulv. alterativ.; while at the same time, I was not deterred from giving small doses of steel, combined, as usual, with a mild aperient. In a few days, calomel and opium were substituted for the alterative powder; but, after a week, the bowels becoming irritable, Plummer's pill was ordered instead. A blister also was applied over the duodenum.

The derangement of bowels being quieted, I deemed it advisable to increase the dose of steel; but as the mixture agreed well, I preferred, instead of changing it, to combine with its use that of the electuary.

The Plummer's pill (I always prefer any name which records individual merit, however slight) was designed to be given only temporarily, until the calomel in a larger quantity, and combined with opium, could be borne; but it agreed so well and acted so favourably, that it was continued up to the 18th of July, when, there being severe tibial pains, with still-existing, though diminished, thickening of the periosteum, recourse was once more had to the calomel and opium. They no longer irritated the bowels; on the contrary, the morbid state of the tongue became daily less marked; the tibial pains at the same time diminished, and gradually ceased altogether; the patient walked with ease, and felt delighted to walk; and her whole general health improved most strikingly. Had it not been for the attack of catarrh, she might have been discharged nearly six weeks sooner than she was.

*Case* 5.—S. H., æt. 23, admitted December 16th.

*Symptoms.*—Strength much impaired; countenance sallow; (although patient is naturally of a sanguineous temperament) pain in both temples, often in occiput; distressing palpitation of heart; tenderness over sacrum with internal sense of pelvic fulness; pain in hypogastric region; bowels open; tongue furred; urine natural, and passed freely; cat. not suppressed, but irregular, scanty, and pale; pulse 110, irritable.

On applying the ear to the chest, the heart was found to pulsate more strongly than natural; but the stroke was quick and sharp, very unlike that of hypertrophy. It was accompanied with a slight bellow's sound.

Illness had existed six months, and had been attributed to diseased heart. I thought very differently of it, and prescribed as follows:

Ext. colocynth comp. gr. v.

Hyd. submur. gr. v.—f. pilulæ duæ h. s. sumend.

Haust. cathart. domest. primo mane.

Purgatione finitâ, sumat ægrota ter de die.

Quinæ sulph. gr. v. necnon haustum sequentem.

Tinct. ferri muriat. ♏xx.

Infusi quassiæ ℥j.—f. haustus.

To prevent a costive state of the bowels, five grains of the pil. hyd. c̄ coloc. were ordered to be taken every night.

Under this treatment, the symptoms rapidly declined; the heart acted more regularly and quietly in proportion as the system became stronger; the bellow's sound ceased to be audible; the patient's complexion became florid; and notwithstanding an attack of influenza which she suffered in the beginning of January, she was discharged "cured" on the 17th of that month, not quite five weeks from the time of her admission.

*Remarks.*—It would be idle to insist at any great length on the necessity of discriminating accurately between affections of the heart resulting from organic disease, and those which are sympathetic, and purely functional. Most, if not all, practitioners are aware that this important difference exists; and it is only from a want of proper attention to the symptoms attendant upon each particular case, that men fall into error, and thereby appear ignorant of the grand general distinction. And yet how often is the one form of disease mistaken for the other! *Inattention,* in fact, is the great source of error in medical practice; and no physician can ever arrive at excellence, who does not, *early in his career,* habituate himself to a strict and minute examination of every case that comes before him. A thorough acquaintance with the general nature of each particular disease will not suffice; there must be a constant habitual exercise of both the perceptive and the reasoning powers; otherwise what is known will not be recognized, and what is recognized will not (as to its individual nature at least) be perfectly understood.

Before dismissing the case, I would draw attention to the sacral tenderness and sense of pelvic fulness, both of which doubtless arose from fæces retained in the rectum; although, at the time, the bowels were (as the patient herself averred, and as the report states) naturally open. The hypogastric pain depended in all probability on the same cause, for it ceased, together with the other two symptoms, on the patient's being well purged.

*Case* 6.—H. C. æt. 16, admitted 17th January, 1837.

*Symptoms.*—Skin of a deep brownish yellow tinge; acute pain in left temple; hurried respiration; præcordial tightness, with turbulent irregular action of heart; pain in right iliac region; bowels irregular; tongue furred; pulse 104, feeble; catamenia have never appeared.

Illness said to have existed only two months.

*Treatment.*—Pulv. ipecac. ℈j. vespere.

Haust. cathart. domest. cras mane, nisi vomitorium inferiorem ventrem expurgaverit.

Jan. 19th. Purgative given—both it and the emetic acted freely—head free from pain.

Pulv. cinchon. flav. ʒss. ter de die, iisdemque temporibus haust. sequent.

Tinct. ferri muriat, ♏xxv.

Infusi quassiæ, ℥j.—f. haust.

Pil. hyd. c̄ colocynth, gr. v. sing. noctibus.

Emetic was repeated with marked benefit on the 24th, and also on the 27th of January.

On the 16th of February a purging draught was prescribed; and, after that day, the patient was directed to take two pills every night, instead of one as heretofore.

On the 22d I find the following report:

Pains all gone; general health greatly improved; natural complexion has returned to cheeks; bowels slow; tongue furred.

Sumat cras mane ol. ricini, ʒj.

Catamenia appeared on the 23rd, and the patient was discharged "cured" on the 28th.

I need scarcely remark that the bark and steel were continued without intermission, from the day on which they were first ordered.

*Remarks.*—The details of this case do not invite to many observations. The patient had, most probably, been ill longer than she herself stated; but yet not long enough for the production of many secondary and complicating symptoms. The case was, indeed, almost a simple, though severe, one of chlorotic fever; and as such readily yielded to the appropriate remedies. Three emetics were prescribed, each with very signal benefit; and purgatives were administered even more unsparingly than usual.

*Case* 7.—M. T. æt. 44, widow, admitted March 21st, 1837.

*Symptoms.*—Great prostration of strength; skin dry and harsh; complexion sallow; lips exsanguious; pain in epigastric and præcordial regions; distressing paroxysms of palpitation, attended, sometimes, with a sense of dissolution; frequent chills, followed by partial flushes; slight œdema of ancles; bowels costive; tongue clean; pulse 110, feeble; catamenia regular but scanty.

Illness commenced eleven weeks since, with a rigor followed by headache; for which she was bled from the arm, being at the time in a state of great debility from a previous attack of influenza.

*Treatment.*—Ol. ricini, ʒj. cras mane.

Quininæ sulph. gr. iij. ter de die, necnon, iisdem temporibus, haust. sequent.

Tinct. ferri muriat. ♏xx.

Infusi quassiæ, ℥j.—f. haustus.

Pil. gambog. comp. gr. v. sing. noctibus.

March 28th.—Palpitations very distressing.

> Præcordiis adhibeatur emp. lyttæ, et, remota cuticula, applicetur pulv. mur. morph. semigranum; superponendo linteum chirurg. non illitum.

The effect of this remedy was not merely to relieve the palpitations temporarily, but to subdue them altogether. They did not at least return during the patient's stay in the hospital.

But, a few hours after the application of the morphia, the head began to feel confused, and heavily painful; and continued more or less so until the first of April, when a blister was applied to the back of the neck. This had the desired effect, and the patient gained strength daily, but being desirous of returning home, she was discharged on the 18th of April, her cure, though far advanced, being not yet complete.

*Remarks.* I shall advert only to two points in the foregoing case, and even to these as briefly as possible. They are—

1st. The age of the patient.

2nd. The effects of the endermic use of morphine.

1st. Chlorotic fever is not often met with in patients above 24 years of age. It does, however, occasionally attack persons more advanced in life; and then it occurs chiefly about the period of the cessation of the menses; and is found to affect mostly unmarried, or widowed females, of a naturally delicate constitution, and whose health has been impaired by distress of mind; by bodily privations; or by some antecedent disease. In such patients, it is generally much more difficult of treatment than in the young.

2. The speedy relief of the most distressing functional palpitations of heart, by the endermic use of morphia is a fact well worthy of attention. I have, in other cases, repeatedly tried the remedy, and always with the same good result. The dose has never been more than a grain, and in general only half that quantity. In no dose whatever will it act so beneficially if administered internally. How are we to account for this difference? That, against a mere external pain, its efficacy should be greater when transmitted through the vessels, or applied to the nerves, of the part, can be easily understood; but that an internal organ should be more influenced by its application to the corresponding external blistered surface, (unconnected though the two be either by vessels or by nerves) than by its administration in the ordinary way—this is by no means so easy of comprehension. We may, it is true, explain it by the "sympathy of contiguity," but what are we the wiser for such an explanation? If any one, however, is satisfied with it, to him in particular the following ascertained fact cannot but be interesting. The application of the morphia to a distant part, (as, for instance, to the temple) has very little effect upon the palpitations, even when we have proof of its affecting the constitution, in the nausea and retching which it produces. And this leads me to another important observation relating to the same point. It is, that the morphia thus applied affects the stomach much more certainly than when the application is made to the epigastric region itself. The fact here stated cannot be said to militate against the doctrine of "contiguous sympathy," unless we altogether lose sight of the influence which the *par vagum* exercises over the stomach.

Among the out-patients some very interesting cases of chlorotic fever presented themselves; but my observations on the disease have already extended to such a length, that I am reluctantly compelled to refrain from a detailed notice of them.

### Fever from prolonged Lactation.

My reasons for ranking this disease among the fevers are the same as those assigned for my classification of chlorosis. I do not expect that they will, at first sight, appear satisfactory to many of my readers; but the more the subject is inquired into, the more, I think, will the justness of my views become apparent. Even minds the most prone to conviction feel, for a time, loth to admit any change in their established notions, be they what they may; but truth and reason are always sure to prevail in the end. If I have these on my side—and I believe I have—there can be no doubt that my opinions will sooner or later come to be generally adopted. But the matter, after all, is not of much practical importance; for as to the treatment of the diseases in question, no difference of opinion is likely to exist.

---

### Dumfries and Galloway Royal Infirmary.

5th August, 1837.

The following statistical report of the cases of typhus fever treated during the time specified below in the Dumfries Infirmary, has been transmitted to us by Mr. G. H. Smith, the house-surgeon of that Institution.

It gives us great pleasure to insert it, and the document reflects much credit upon Mr. Smith, who drew it up, as well as on the medical officers who have sanctioned it. We will gladly open our columns to such clinical reports, and we are sure that if the physicians and surgeons of the various excellent Provincial Hospitals will encourage them, the publication will redound to their own honour and advantage, as well as to the interests of science. We have said more on this subject in another place.

### Report.

The whole number of cases treated from the 1st of February to the 1st of August of the present year, amounted to 109; of that number 15 proved fatal, *i. e.* 1 in $7\frac{4}{15}$. Of these there were males 54; and females 55; the mortality of the former, was 1 in $6\frac{3}{4}$, and in the latter 1 in $7\frac{6}{7}$.

The disease was traced to contagion in 59 cases, at least, the whole 59 cases were either persons of the same families and living in the same houses, or persons living in infected localities, and in the habit of visiting those ill with fever. In the remaining 50, it was traced to various causes, but chiefly to exposure to wet and cold, to living in ill-ventilated apartments, and to deficient and unwholesome food; but in a few cases no cause could be assigned.

The day of the disease on which the patients were admitted varied from the first to the fourteenth day; but the majority from the fifth to the seventh day; and the whole averaged the seventh day.

The day of the disease on which the crisis was observed varied very much; it most frequently happened on the 11th, 13th, 15th, 17th, and 21st days; in some cases it was well marked; in others it was very obscure.

The following Table exhibits the number of cases treated between the different ages as marked; the average day of the disease on which they were admitted, the average day of the crisis, the average time in the hospital, the number in which the rosy eruption, petechiæ, or both eruptions, or in which no eruption was observed; the parts to which the fever determined, and the result of the cases. It also shows the proportion of deaths and cures, where an eruption or where no eruption was a symptom, and also the proportion under the different heads, local affection; and likewise the average day of admission, crisis, and period in hospital, under each complication. The cases which proved fatal, are not taken into account in striking the average for admission, crisis, and time in hospital.

[*See Table next page.*]

From the Table the following inferences may be deduced.

First. That the most frequent form of fever here is where there is evidence of disorder in the head, chest, and belly at once; and differing little from this in point of frequency are those cases in which the head and belly alone are affected. The former form occurred in rather more than one-fourth, and the latter in nearly one-fourth, of the whole number of cases. The next most frequent form is found where there are symptoms of disturbance in the head, with some local affection in the chest, and that generally of the bronchial mucous membrane; and the same number of cures is found where there were signs of abdominal disease alone. Both complications occurred in rather more than one-eleventh of the cases.

Second. That the lagest proportion of fatal cases is found to be, 1st, in those cases in which there were the combination of symptoms referrible to the head, throat, chest, and belly—three, or one-half of the whole cases having proved fatal; 2d, those in which there was determination to the head, chest, and belly; in which six, or rather more than one-fifth of the cases proved fatal—and constituting two-fifths of the whole mortality. And lastly, in those in which there was diarrhœa with local affection in the head and belly. Of this class of cases one-sixth terminated fatally.

Third. It appears that petechia, and more particularly when present with the rosy eruption, is a most unfavourable symptom; no less than one-half of the whole cases having proved fatal, and forming more than one-half of the whole mortality.

Fourth. That the most protracted cases were, 1st, those classed under local affection of the head, throat, chest, and belly; 2nd, under the head and belly, with diarrhœa; and to the same degree were those in which there were symptoms of disorder in the head and chest.

Fifth. That the greatest number of cases was admitted between the ages of 10 to 20 and 20 to 30; the rate of mortality least between the ages of 10 to 20; and if we except the ages of 50 to 60, where all died, the rate of mortality increased with the increase of years.

For more minute particulars reference must be made to the Table; but it may be remarked that those complications, where the largest proportion of deaths is found, are not invariably the most protracted forms of fever.

| AGE. | Males. | Females. | Total treated. | Average Day of the Disease admitted on. | Average Day Crisis took place on. | Average Time in Hospital. | Rosy Eruption a Symptom. | Petechia a Symptom. | Both Eruptions Symptoms. | No Eruption. | No Local Affection. | Local Affection in the Head. | Local Affection in the Chest. | Local Affection in the Belly. | Local Affection in Head, and Parotids. | Local Affection in Head and Chest. | Local Affection in Head and Belly. | Local Affection in Head and Belly, with Diarrhœa | Local Affection in Chest and Belly. | Local Affection in Head, Chest, and Belly. | Local Affection in Head, Throat, Chest, and Belly. | Local Affection in Throat, Chest, and Belly. | Cured. | Died. | Local Affections of the Cases which terminated fatally. |
|---|---|---|---|---|---|---|---|---|---|---|---|---|---|---|---|---|---|---|---|---|---|---|---|---|---|
| Under 10 | 5 | 5 | 10 | 6 | 13 | 17½ | 3 | 1 | .. | 6 | 2 | 1 | 1 | 2 | 1 | .. | 3 | .. | .. | .. | .. | .. | 9 | 1 | Head and Parotids. |
| 10 to 20 | 12 | 19 | 31 | 6 | 14 | 18¼ | 14 | 3 | 1 | 13 | 3 | 2 | 1 | 5 | .. | 2 | 6 | 2 | .. | 6 | 1 | 3 | 29 | 2 | Head, Chest, and Belly; and Head, Throat, Chest, and Belly. |
| 20 to 30 | 15 | 15 | 30 | 5 | 16 | 22 | 16 | .. | 1 | 13 | 1 | .. | .. | 2 | .. | 3 | 7 | 1 | .. | 10 | 5 | 1 | 27 | 3 | Head, Throat, Chest, and Belly in two; and Head and Belly in one. |
| 30 to 40 | 13 | 5 | 18 | 6 | 15 | 15 | 7 | 1 | 1 | 9 | .. | 4 | .. | .. | .. | 3 | 3 | 2 | 1 | 4 | .. | 1 | 16 | 2 | Head, Chest, and Belly; and Head and Belly, with Diarrhœa. |
| 40 to 50 | 6 | 7 | 13 | 7 | 16 | 22¼ | 4 | 3 | 1 | 5 | .. | 1 | .. | 1 | .. | 1 | 3 | 1 | 1 | 5 | .. | .. | 11 | 2 | Head, Chest, and Belly. |
| 50 to 60 | 1 | 2 | 3 | .. | .. | .. | .. | 2 | .. | 1 | .. | . | .. | .. | .. | .. | 2 | .. | .. | 1 | .. | .. | .. | 3 | Head, Chest, and Belly in one; and Head and Belly in two. |
| 60 to 70 | 2 | 2 | 4 | 12 | 22 | 16 | 1 | .. | 2 | 1 | .. | .. | .. | .. | .. | 1 | 1 | .. | .. | 2 | .. | .. | 2 | 2 | Head and Chest; and Head, Chest, and Belly. |
| Total .... | 54 | 55 | 109 | 7 | 16 | 18½ | 45 | 10 | 6 | 48 | 6 | 8 | 2 | 10 | 1 | 10 | 25 | 6 | 2 | 28 | 6 | 5 | 94 | 15 | |
| Deaths ... | 8 | 7 | 15 | .. | .. | .. | 4 | 4 | 4 | 3 | .. | .. | .. | .. | 1 | 1 | 3 | 1 | .. | 6 | 3 | .. | .. | 15 | |
| ures.... | 46 | 48 | 94 | 7 | 16 | 18½ | 41 | 6 | 2 | 45 | 6 | 8 | 2 | 10 | .. | 9 | 22 | 5 | 2 | 22 | 3 | 5 | 94 | .. | |
| Average day of admission........ | | | | | | | 6 | 7 | 9 | 6 | 4 | 5 | 8 | 4 | .. | 9 | 6 | 7 | 4 | 8 | 8 | 6 | 7 | .. | |
| Average day of crisis............ | | | | | | | 17 | 15 | 17 | 13 | 9 | 11 | 16 | 11 | .. | 19 | 14 | 19 | 14 | 17 | 21 | 17 | 16 | .. | |
| Average time in hospital under each complication | | | | | | | | | | | 14 | 13½ | 12 | 15 | .. | 22 | 18½ | 27½ | 17 | 22 | 24 | 19 | 18½ | .. | |

The following circumstances may not be uninteresting, viz. that in one case the patient was under the influence of mercury for the cure of a venereal complaint when seized with the fever—the case terminated fatally; in six cases the catamenia appeared with the invasion of the disease—two of which cases proved fatal; in one case abortion happened between the third and fourth month—the patient recovered; in two, epistaxis took place during the progress of the fever—both recovered, but one of the cases made a very protracted recovery; in this case blood likewise oozed from the gums, and the urine had a bloody tinge.

The day of the fever on which death took place varied from the 10th to the 31st, and averaged the 15th day. Of the 15 fatal cases, permission for a post-mortem examination of 11 was obtained; it was performed from 12 to 36 hours, but generally 24 hours, after death. The following Table exhibits the morbid appearances.

| | |
|---|---|
| Inflammation of membranes of brain in .. . .. .. .. | 2 |
| Venous and arterial congestion in head in .. .. .. .. .. | 8 |
| Effusion of serum beneath arachnoid in .. .. .. .. .. | 9 |
| Effusion of serum between layers of arachnoid and into ventricles in .. .. .. .. .. | 3 |
| Serous cyst in posterior lobe of right hemisphere in .. .. | 1 |
| Deposition of bone between the layers of falx cerebri in .. .. | 1 |
| Inflammation of pleura acute in, | 2 |
| Effusion of serum into pleural cavities in .. .. .. .. | 2 |
| Recent adhesions of pleura in 1 and old adhesions in 4 .. .. | 5 |
| First degree of hepatization of lungs in 1; and third degree of hepatization of lungs in 1 .. | 2 |
| Bronchitis acute in .. .. .. | 5 |
| Ulceration of larynx and œdema of epiglottis in.. .. .. .. | 1 |
| Opaque spots on right auricle of heart in .. .. .. .. .. | 1 |
| Ossification of mitral valve, and contraction of corresponding aperture in .. .. .. .. | 1 |
| Congestion of mesenteric veins in | 11 |
| Adhesions (old) of the omentum and mesentery to abdominal parieties in .. . .. .. | 2 |
| Glands of Peyer enlarged in .. | 6 |
| Isolated follicles of great intestine enlarged in .. .. .. | 4 |
| Softening of mucous membrane of small intestine in .. .. | 3 |
| Ulcerations of small and large intestine in.. .. .. .. .. | 1 |
| Intus-susceptions of small intestine .. .. .. .. .. .. | 2 |
| Congestion & softening of spleen in 2; and granular appearance of in 1 .. .. .. .. .. | 3 |
| Inflammation of mucous membrane of bladder in.. .. .. | 1 |
| Hypertrophy of substance of uterus in 1; and ulceration of neck of ditto in 1 .. .. .. .. | 2 |
| Adhesion of uterus to peritoneum lining pelvic cavity in .. .. | 1 |

The most constant appearances were as follows:—In the head, venous and arterial congestion, and effusion of serum between the arachnoid and pia mater. In the chest, marks of acute bronchitis. In the abdomen, congestion of mesenteric veins, and enlargement of Peyer's glands. The glands were most frequently bound at the lower end of the ilium in patches of an oval shape, varying from an inch to several inches in length.

G. H. SMITH, House Surgeon.

### Gleanings during Three Years spent in the Indian Seas.

### Swan River and King George's Sound.

For the following sketch, we are indebted to our friend Mr. William Barrett Marshall. Our readers will probably remember a notice of the medical establishment of the British Auxiliary Legion in Spain. This was from the same pen. Mr. Marshall, now one of the surgeons of the Kensington Union, has seen much in the Royal Navy of disease abroad, and has not only seen but observed it. We hope we shall be enabled on many occasions to introduce him to our readers.

*Nosological Report, for the Town of Fremantle, in the Colony of Western Australia, from the Commencement of the Colony in* 1828, *to December* 1833.

| DISEASES. | Number of Cases. | Recovered. | Died. | REMARKS. |
|---|---|---|---|---|
| CŒLIACA. | | | | |
| Entrrica. | | | | |
| Dysphagia Mulosa .. | Six. | Six. | | |
| PNEUMATICA. | | | | |
| Pneumonica. | | | | |
| Bex Convulsiva ..... | Unknown. | Unknown. | Unknown | No case of this disease occurred in the Colony until after the arrival of the 21st Fusiliers. The Children belonging to the detachment were affected on their arrival. A few of the cases proved fatal at Perth, but none at Fremantle. |
| HÆMATICA. | | | | |
| Pyrectica. | | | | |
| Auctus Tertiana .... | Four. | Four. | .... | The subjects of this complaint were all sailors, who arrived, sick, in the Colony. |
| PHLOGOTICA. | | | | |
| Apostema Empyema .. Empresma | Six. | .... | Six. | The patients were Lascars, and brought the disease with them from India. |
| *Cephalitis* ....... | Twenty-six. | Twenty-six. | .... | The attack was, in every case, slight; and yielded readily to the antiphlogistic treatment. |
| *Pneumonitis* ..... | Four. | Four. | | |
| *Gastritis* ........ | Two. | Two. | | |
| *Enteritis* ........ | Three. | Two. | One. | In two out of the three cases, the sufferers had been inmates of the Penitentiary at Millbank, and, while there, were attacked with the epidemic dysentery that prevailed. |
| *Hepatitis* ........ | Five. | Five. | | |
| Ophthalmia. | | | | |
| *O. Purulenta* .... | .... | .... | .... | The number of cases was not to be obtained; every man in the Colony being said to have experienced the disease once at least. In a few instances, it has recurred a second time in |

| DISEASES | Number of Cases. | Relieved. | Died. | REMARKS. |
|---|---|---|---|---|
| Dysenteria. D. Acuta ........ | 1 Hundred and one. | Ninety-five | Six. | the same individual;—the greatest sufferers were those of intemperate habits From Sept. 27th, 1829, to Nov. 20th, 1833—average duration of the complaint, ten days—blood-letting indicated in only four cases. |
| DYSTHETICA. Marasmus Tabes.... | Three. | .... | Three | One of these cases was that of an invalid from India, the others were those of individuals who had the complaint before they came to the Colony. |
| Porphyra Simplex .... | Forty. | Unknown. | .... | Characterized by soft, spongy bleeding gums; and aggravated by contractions of the joints. It commenced with the arrival of the first settlers, but is now a rare disease, not a case having occurred for 18 months; it proved fatal in numerous instances. |
| NEUROTICA. PHRENICA. Ecphronia Mania ... | Six. | Two. | One. | The death was suicidal. Two of the cases were removed from the Colony, and one is still afflicted. |
| ÆSTHETICA. Neuralgia faciei .... | One. | .... | .... | |
| manus .... | One. | One. | .... | This case yielded to a course of dietetic and alterative remedies. |
| SYSTATICA. Carus Apoplexia.. .. | One. | .... | One. | |
| ECCRITICA. CATOTICA. Paruria Retentionis.. | Fifteen. | Fifteen. | .... | Ascribed to water-drinking, and dram-drinking both—the water of the place being very brackish; and the greater number of patients, sailors, of alleged intemperate habits. |

The data from which the foregoing Report has been drawn up were supplied me by the courtesy and kindness of Mr. Harrison, Assistant Colonial Surgeon. They refer almost entirely to Port Fremantle, and the district within which it is situated. Further particulars I failed to obtain from the Colonial Surgeon who resides at Perth, the capital of the territory—and this report, therefore, only conveys a partial view of the "ills to which flesh is heir" in the new settlement of Swan River.

The cases of dysentery and scurvy had their origin in privation and want, the earlier settlers having had more than an ordinary share of difficulties to contend with, and having been peculiarly improvident, or unfortunate, or, perhaps, both.

Here, as everywhere else, among civilized men, inebriety has added its multitude of victims to the sick-list—and aggravated the diseases of others—and, in many more instances, indisposed the system to receive benefit from medicine where the disease itself has not arisen from intemperance. Many of the cases of paruria retentionis, empresma cephalitis, and ophthalmia, were examples of this.

But, although the ophthalmia which so universally spread among the settlers, assumed an aggravated character when occurring in persons of intemperate habits, its universality must be accounted for in some other way—and to do this is not difficult. Port Fremantle is built on a small isthmus, the sides of which are washed by the waves of the sea on the one hand, and the waters of "the Swan" on the other. The soil is a white sand, glistening with particles of rock-salt, and crystal, and constituting a mirror from which the glare of the sun is reflected upon the eye of every settler; the houses are, for the most part, built of lime-stone. The winds which set in, alternately, now hot from the land, and now cold from the ocean, lift up the finer grains of sand, and convey them over miles of sea and land by turns—these, touching the eye, irritate, and irritating, inflame. And these, being causes continually present, and universally applicable, will sufficiently account for the prevalence of so painful an affection, in the case of almost every settler in the Colony.

The temperature varying from the freezing point of Fahrenheit to 106°, and frequently differing more than twenty degrees in the same day—and the climate subject to sudden changes, the surprise is that there have happened so few cases of rheumatism. During H. M. S. Alligator's stay at Swan River, December, 1833, the thermometer ranged from 70° to 105° in the shade; the evenings and mornings being much colder than the intermediate portions of the same day. A hot land-wind prevails during the forenoon—a refreshing sea-breeze from the *cold* South sets-in in the afternoon; the latter is equally conducive to the health and convenience of the inhabitants, cooling the air they breathe, and facilitating their commerce with the interior by means of the principal river.

The numerous cases inserted in the report as cases of empresma cephalitis, can hardly, I should think, deserve so bad a name. I suspect they were cases chiefly of cephalia pulsatilis—arising from exposure to the sun, and principally occurring in persons of drunken habits; the symptoms being, sudden and violent headache, flushed face, vertigo, and sickness; but without delirium; continuing not longer, upon an average, than two or three days—and readily yielding to venesection, purgatives, and the application of refrigerant lotions to the head.

The above may serve to convey a tolerable estimate of the state of health in Western Australia, although a register only of diseases occurring in a single locality. Perhaps the modifications of disease in other places is slightly in favour of the inhabitants—as at Perth, the capital of the Colony, where the presence of the Government exerts a salutary influence over the habits of the settlers recommending temperance by its example, and repressing excess by its influence.

There is a colonial surgeon at Perth, A. Collie, Esq. R.N., with a salary of £200 per annum, and, of course, the private practice of the whole of the respectable families located there. There is also an assistant surgeon at Port Fremantle, Mr. Harrison, who has the responsibilities of a surgeon, and the care of the gaol; with the duties of quarantine to attend to besides. Medicines are provided at the Government expense. A room has been fitted up at Perth for the reception of patients, and is sufficiently roomy during the infancy of the settlement, and the paucity of sick poor.

At King George's Sound, Mr. Lyttleton is the Colonial Assistant-Surgeon, with the pay and allowances of an assistant-surgeon in the Army—and at Port Augusta there is a third medical officer, with an allowance of five shillings per diem.

Besides these four medical men, who are permanently-attached to the Colony, there were, in December 1833, two others, assistant-surgeons in the Army,

Messrs. Davidson and Milligan, who accompanied detachments of His Majesty's 21st Fusiliers, and 63rd Regiment. There was also a private practitioner at Fremantle, who divided the practice of that place with the official one. Two others are said to be living elsewhere, not so much upon their professional earnings, as by their skill in agriculture—to which they have been obliged to turn for that daily bread which the practice of medicine failed to secure to them.

At King George's Sound, I requested Mr. Lyttleton's assistance to draw up an account of the diseases of Albany—and am indebted to him for the concise yet comprehensive intelligence, that all the diseases which have befallen the settlers there, had their origin in privation, or in excess. A testimony which speaks volumes for the climate.

*Extract from the MS. Journal of W. B. Marshall, R.N., Author of "A Personal Narrative of Two Visits to New Zealand." London,* 1836. *Nisbet.*

### NORFOLK ISLAND.

On the wide-spread surface of our globe there is perhaps no one spot of earth, where misery is rifer, or crime more abundant, than on the above island. At the time of my visit to it, in 1834, there were seven hundred and fifty prisoners, a hundred soldiers, and about fifty civilians. The whole island is, at one and the same time, a garden and a gaol. Vegetation is luxuriant in the extreme, and the surface of the country so diversified, that at every turn the eye pauses to enjoy scenes of natural beauty, unsurpassed by those of any other land. Earth, air, sea, and sky, are here all alike beautiful and salubrious. But man, in the emphatic language of one of the condemned malefactors, is man no longer here;—the natural wilderness is indeed a garden, but the moral garden is a melancholy desert, on which no flower is suffered to flourish, no fruit to ripen.

Of the Prisoners on the island, about two hundred had been capitally convicted at Sydney, constituting what is termed the respite gang. The remainder had all been convicted of crime in New South Wales, after having been sent from England under sentence of transportation. And almost every man on his arrival gives himself up to the recklessness of despair, considering his character irrevocably damned, and himself abandoned to utter wretchedness and everlasting shame. Under circumstances like these, it might be imagined that the depressing passions would affect the health of the prisoners, and more than counterbalance the salutary influence of military discipline, wholesome restraint, compulsory temperance, and the most healthy climate in the world. Not so, however, for there is an apparent elasticity in crime itself, which not only resists every attempt to overcome it, but has power to lift the chained and branded felon out of all sense of shame into a condition of heartless indifference and even joyousness. It will not therefore be matter of surprise, that there are more cases of counterfeit than of real disease—and that the average number of deaths by disease and casualties did not exceed nine annually, for three years together not more than one per cent.—of which, there were four by the hand of the executioner, and two by that of the murderer; while three men were shot, one in attempting to escape with a boat, and two accidentally—one was accidentally killed, and three more were drowned. Fifteen out of twenty-seven deaths, in three years, happening by other means than disease, reduce the proportion to less than one half per cent. per annum. The daily average of sick, during the year 1833 did not exceed 31, or about one in thirty.

There is, nevertheless, an assistant-surgeon attached to the settlement, and also an assistant-surgeon attached to the troops. The former has the sole charge of the convict population and of the civil establishment—the duties of the latter being confined to the military.

There is also a small Hospital, with accommodations for forty patients, divided into five wards, a dispensary, receiving-room, dead-house, store-room, overseer's room, and cook-house. One of the wards, (all of which were very clean, and in excellent order, at the time of my visit), is appropriated for the reception of patients from the gaol gang, as that portion of the prisoners is denominated, who, in addition to the crimes for which they were sent from England, and the crimes for which they were re-transported from New South Wales, have committed others on the island, and been punished with confinement to the gaol.

The Hospital establishment consists of an Assistant-Surgeon, with the pay and allowance of an Assistant-Surgeon in the army—and, under him, a dispenser and overseer, a waterman, a wardsman, and a cook; all of whom are convicts.

When I inspected it, there were only seven patients in the Hospital, and none of them wholly confined to bed. Besides them, there were a dozen out-patients who attend daily, for advice and medicine.

The men are dieted according to a prescribed scale, by daily direction of the medical officer, who varies it at his pleasure.

A diet list is hung up in every ward, alongside of a large board, on which are painted, as follow, the Commandant's orders:—

1st. No person whatever is to be admitted into the Convict Hospital, without permission from the medical officer.

2nd. Any officer's servant, or other person, going there to receive medicine, or on any other business, to stand outside the gate.

3rd. No orderly, or other attendant in the Hospital, is, on any account, to quit it without permission of the medical officer.

4th. The patients are not permitted to go either into the kitchen or the orderly's room, or any room but the ward to which they are appointed.

5th. The orderlies, patients, and all persons attached to the Hospital, are to receive and strictly attend to all regulations and rules, that the Assistant-Surgeon may, from time to time, think necessary to institute.

6th. The gates of the Hospital are to be kept constantly locked.

7th. The overseers and orderlies are held responsible that these orders, as well as all others that may be given, are strictly obeyed.

8th. To insure the patients their diet, and what may be ordered for their comfort by the surgeon, and to prevent its being withheld by the attendants, a diet roll will be put up in each ward of the Hospital every morning, stating what has been ordered by the Assistant-Surgeon, that day, for each patient.

Nosological returns are sent in to Head Quarters at Sydney, for the use of the Colonial Government, monthly and quarterly.

---

### Guy's Hospital.

Guy's Hospital Reports. No. 5, October, 1837. Edited by G. H. Barlow, M. D., and J. P. Babington, M. A.

The contents of this number are neither few nor unimportant. The School of Guy's Hospital has already, if we are not misinformed, derived some benefit from the publication of these reports. And so it should. We trust the time is passing by, when either pupils or practice will be obtained by the influence of puffing or party, by the humbug of a name, or the still greater humbug of a selfish and a spurious liberality.

The Schools of London, attached to the considerable Hospitals, are making great exertions to maintain, and to advance their characters. Their matériel is enormously increased—the mode of teaching absolutely revolutionized—and the teachers themselves, under the operation of competition and necessity, are making unparalleled exertions. Could the most popular lecturers of twenty years ago revisit a large metropolitan class-room, they would admit that, at all events the system was improved, whatever they might think of their successors personally.

Among the main advantages of a large school, we must place its connexion with a considerable hospital. From *it* emanates the instruction to be obtained both from the living and the dead. However excellent lectures may be, the student does not and cannot acquire his highest and most useful knowledge from them. He must have the means of dissecting the dead—of noticing and of personally cultivating the important and extensive facts of morbid anatomy—and, above all, he must have the opportunity of observing disease on a large scale. His lectures are only an introduction to the wards—if he cannot enjoy the advantages that they offer, he had best:

"Break his pipe and never whistle mair."

To display the facts which the student of nature may witness in Guy's Hospital, has been the object of its medical officers. Their reports have been continued with regularity, and conducted with ability and zeal. The volumes they will form are sure to prove a valuable addition to the library of a practical man. We trust that the medical officers of the other hospitals to which large schools are attached will emulate the example set by the physicians and surgeons of Guy's. We do not insist on the publication of reports in a distinct work. That plan has both advantages and inconveniences. But we do urge the regular publication of reports, in such a form as to ensure to the profession an acquaintance with the facts, which would else be all but buried in the institutions that contain them.

The part of the Reports of Guy's Hospital before us comprises the following papers:—

1. Observations on the Ganglionic Enlargement of the Pneumo-Gastric Nerve, &c.; by Mr. Edward Cock.
2. Observations and Experiments on Lungs of New-born Children, in relation to Medical Jurisprudence; by Mr. Alfred S. Taylor.
3. Cases of Gangrene, Aneurysm, Ununited Fracture, Hernia, Wound of the Tongue, and Stone in the Bladder; by Mr. Bransby Cooper.
4. On the Diagnosis of Organic Diseases of the Uterus; by Dr. Ashwell.
5. Observations on Abdominal Tumors, and Intumescence; illustrated by cases of Acephalocyst Hydatids; by Dr. Bright.
6. On the Influence of Electricity, as a remedy in certain Convulsive and Spasmodic Diseases; by Dr. Addison.
7. Case of Disease in the Fœtus; by Mr. T. W. King.
8. On the Distribution and probable Function of the Superior and Recurrent Laryngeal Nerves, as demonstrated, by dissection, in the Human Subject; by Mr. John Hilton.
9. Description of the Sacculus or Pouch in the Human Larynx; by Mr. John Hilton.
10. Account of the successful termination of a case of Sphacelated Intestine and Omentum in a Femoral Hernia; by Mr. Aston Key.
11. Mr. Morgan's Ophthalmic Cases.
12. Experiments and Observations on Albuminous Fluids; by Dr. Babington.

We shall select for notice in this article, the cases and observations of Mr. Bransby Cooper. The Anatomical and Physiological papers will be considered in connexion with some recent anatomical works.

### Cases of Gangrene, Aneurysm, &c. By Mr. Bransby Cooper.

A. *Dry Gangrene of the Hand.*—Samuel Harlow, aged 18, had never been strong, and had for some years suffered, in the Winter, from cough, attended with expectoration slightly tinged with blood. He always required good diet, and took meat and a pint of porter daily.

About three weeks before his admission, whilst planeing a piece of wood, he struck, with considerable force, the end of the nail of the right ring or third-finger, against a piece of lead placed upon the wood to steady it. This blow gave him pain at the root of the nail, shooting up the arm: after a short time, however, it became comparatively easy, and did not interfere with his work for more than a few minutes. On the following day, the root of the nail, and the skin for about a quarter of an inch above that point, had a bruised appearance, and felt cold and numbed, with little pain in the part; no swelling, pain, or tenderness up the arm or in the axilla; and he continued his work as usual, feeling himself quite well. He did not at the time apply any thing to the finger. This bruised or ecchymosed appearance, and the other local symptoms, did not appear to extend; but on the third day from the accident, the palmar aspect of the fore and middle fingers became gradually numbed, cold, and shrivelled, and whiter than the healthy fingers; and also very tender and painful, the pain being confined to the end of the fingers. The ring-finger, the original seat of the injury, now became affected above the parts which had the bruised appearance, and seemed inflamed as far as the second joint. In about a week, these local symptoms (without deranging his general health) extended, in all the fingers, as far as the second joint. At this time, when immersed in warm water, they lost their pale or white appearance, and became blue and more painful. They again resumed their death-like appearance, upon removing them from the artificial heat.

Four days before his admission, the thumb became affected as the fingers had been, and next day the little finger was affected too.

On Oct. 7, he was admitted into the Hospital. His aspect is exsanguined and depressed—the whole body cold—tongue furred—pulse at the left wrist 92, and sharp—pain in the right arm below the elbow and hand.

The fingers and thumb present a livid hue, extending up the palm of the hand: this livid aspect is not intense at the extremities of the 1st, 2d, and 3d fingers, but at the other parts. The skin has a deep-mottled appearance; the fingers are cold as high as the first phalanges, and swollen; but the end of the finger which received the original injury is shrivelled and black, and in the condition of dry gangrene. He has no sensation on the dorsal aspect of the hand below the first phalangeal articulations. The sensation of the parts supplied by the cutaneous branch of the median remains pretty good, but no where so good as in the other parts of the body. Below the palm, the sensation is lost, except on the little and ring finger, where pressure may be felt. The brachial artery, at the upper part, feels somewhat cord-like, apparently from the thickening of its coats; and, on tracing it downwards, it becomes more so; and the pulsation, after gradually diminishing, ceases at the elbow, about two inches above its division into ulnar and radial, where it appears solid, without pulsation. The solid condition may be traced along the two first inches of the radial artery, where it disappears; but it is again recognised about three inches above the wrist; and from that point continues so, throughout its distributions, as far as they are tangible. No pulsation can be felt in the ulnar artery: it also feels solid from below the wrist, to about three inches above: and just above where the solidity terminates, he complains of severe pain when the course of the artery is pressed upon.

On examining the right subclavian, about an inch to the outside of the scalenus, there is a rough sawing noise audible. A small enlargement, probably a gland, is felt under the vessel.

The treatment prescribed was good diet—local warmth—aperients—and narcotics.

The gangrene extended slowly, and the cessation of pulsation in the brachial artery was noticed higher. There was much pain in the hand. On the 20th of October, the cuticle, just above the two first metacarpo-phalangeal joints had become greenish. On the 22nd, a line of separation had commenced. On the 27th a similar line was discoverable in the palm. On the 2nd of November granulations had sprung up. On the 11th, Mr. Cooper removed the hand at the radio-carpal joint. No arteries required to be tied, as the radial and ulnar were filled up with coagula; although the anastomosing branches bled sufficiently freely to lead to the hope that the stump would heal.

The stump suppurated, but ultimately did well. On December 6, upon careful examination, the profunda artery appears very much enlarged, and may be distinctly felt pulsating; and the radial also has resumed its function, although the pulsation in it cannot be felt so distinctly as in the left wrist.

He was discharged from the hospital, quite cured, on the 6th of January 1837.

The simplest explanation of this curious case, is, perhaps, the following:—that local injury excited inflammatory action which in a person of a peculiar and enfeebled constitution terminated in gangrene. What share the pressure of the enlarged gland in the axilla upon the subclavian artery exerted in the production of the phenomena which were observed, it is difficult to determine. Mr. Cooper is of opinion that:—

"Some slight degree of absorbent inflammation extended up the arm, and led to glandular enlargement in the axilla and above the clavicle: in the latter position, a tumor, like an enlarged gland, was soon discovered behind, but pressing upon the subclavian artery, which in itself might have been sufficient to lead to inflammation of its internal coat, the effusion of lymph, and its ultimate obliteration. It is difficult to believe that inflammation of the artery should extend from the hand up to the axilla, without much more constitutional irritation, and the loss of the whole limb from sphacelus; for, in that case, the obliteration of the vessels would have extended successively from below upwards; while, on the contrary, here the cessation of pulsation was from above downwards: and therefore we have more reason to believe that the disease in this boy was induced by pressure upon the artery, rather than by any specific disease of its internal coat; although, perhaps, it cannot be denied, from the peculiarity of his circulation, that there was some constitutional tendency to sub-acute arteritis."

B. *Ununited Fracture—Influence of Mercury.*—A healthy-looking young woman, aged 28, was admitted, March 9, 1836, with an ununited fracture of the left humerus, just below the deltoid muscle.

Six months previously, when in good health, she was thrown from a cart, and received the fracture. Splints were applied, but at the end of eight weeks there was no union. The splints were replaced, and the arm bound to the side for a month. There was still no union. The apparatus was replaced with the addition of an iron splint, extending from the outer side of the humerus to the wrist, being bent at an acute angle: and the arm was, as before, bound to the side.

This plan was also persisted in for a month, but with no better effect. The surgeon, still unwilling to give up the case in despair, made one other attempt, by applying a bandage tightly round the arm; and, placing wooden splints over it, compressed them to the utmost the patient could bear, to which she submitted patiently for two months; at the expiration of which period, the fractured extremities of the bone were found as moveable as ever.

On admission, the two portions of bone moved readily upon each other, but without producing any thing approaching to the sensation of crepitus: on the contrary, the mobility of the part conveyed the impression of the formation of a supernumerary joint, and the muscles were capable of producing some voluntary motion. Mr. Cooper proposed the introduction of a seton between the ends of bone. This was done on the 23rd of March. On the 25th, there was some fever, and local irritation. But the seton failed, after a trial of ten weeks. Then a bandage dipped in a composition of egg and flour was worn—then the limb was enveloped in plaister of Paris. Neither plan succeeded.

"About this period, Mr. Collis, from Dublin, paid a visit to Guy's Hospital; and, in going round the wards, Mr. Cooper drew his attention to the case in question. He said he had seen the administration of mercury, continued until it produced ptyalism, lead to the consolidation of ununited fractures, after all other means had failed; and quoted two cases in illustration of the assertion. The patient was accordingly immediately ordered four grains of Hyd. c̄ Cret. three times a-day; and a well-padded leathern girth, furnished with straps and buckles, was firmly applied immediately over the seat of fracture. In four days, ptyalism was produced, and the quantity of mercury was diminished. On the sixth day the powders were suspended, as she suffered severely from their effects. The leathern girth was worn a month; and upon its removal, perfect union of the bone had taken place; affording satisfactory proof that the mercury had produced an altered action on the capillaries of the affected part, and exemplifying the powerful alterative influence of that metal. She remained in the Hospital six weeks after this happy result, to regain her strength: when she was discharged as cured, and with a perfect use of her arm.

"Three months after her departure, she was again admitted into Guy's Hospital, for a fracture of the same arm, produced by a violent blow, inflicted by a man running with great velocity against her, and knocking her down. Upon examination, it was found that the humerus was fractured rather below the seat of the former injury: all the usual concomitant symptoms of simple fracture were present, as crepitus, &c.; and by the application of the same girth which had been employed on the former occasion, the bone united at the usual period, without the exhibition of mercury."

c. *Fatal Hæmorrhage from Wound of the Tongue.*—A strong young man, aged 20, was admitted Sept. 4. He stated, that, three days before, he was smoking with a fellow-workman, when, by accident, the elbow of his companion came in contact with the bowl of his pipe with great force, and drove its narrow extremity into his tongue, breaking off about three inches of the pipe: he immediately sat down upon some steps, suffering excruciating pain, and fainted away. At the time of admission, a wound was perceptible, passing obliquely through the tongue, from the right to the left side. There was no hæmorrhage from the wound; but the tongue and fauces were much swollen. A swelling of considerable size existed, just behind the angle on the left side of the jaw. A surgeon had examined the wound, immediately after the accident, who could not discover that any portion of the pipe remained in the tongue; and this was also the patient's opinion. Leeches were applied to the swelling, and a cathartic draught was prescribed. The following day he seemed much relieved; and already the wound in the tongue had closed. On the 5th, the tongue could be drawn into the mouth; and his speech and deglutition were much freer.

*Sept. 6.* He discharged from the nose and mouth about a pound of blood; which was a dark coagulum, and appeared to be thrown off from the stomach. He seemed much reduced by this loss of blood, which recurred on the 8th and 10th of September. On the 12th, he had become excessively reduced; and might be said to be in a perfect state of anæmia. No wound could be discovered in the tongue, or any where in the mouth, to point out from whence the hæmorrhage proceeded. There was, however, so much swelling about the tongue and fauces, and the jaw could be but so imperfectly depressed, that only a very unsatisfactory examination could be made of the parts, and the source of the bleeding still remained in obscurity; nor was there any evidence of the presence of a foreign body in the inflamed parts.

Plumb. Acetat. gr. ¾, Pulv. Opii gr. ½. M. ft. pil. quâque quartâ horâ sumend.

Acid. Sulph. dil. gtt. xv. Inf. Rosar. ℥iss. M. ft. haust. ter die sumend.

14. He appeared to be sinking fast; and ejected a considerable coagulum from his stomach during the visit; which reduced him to so low a state, that transfusion was contemplated, but relinquished in consequence of the patient's now rallying in the afternoon. Two grains of the sulphate of quinine were ordered to be added to the infusion of roses and acid, and an alum gargle directed to be employed.

15. He died at seven P. M. almost immediately after a discharge of blood from the mouth, which was still brought up as a coagulum from the stomach; although there never was any bleeding into the mouth, as if from thence the blood issued.

*Dissection.* The surface of the body was in an exsanguined state. On injecting the carotid arteries with fine injection, the coloured fluid flowed copiously from both nostrils, particularly from the left. A small irregular opening was discovered, just behind and below the left tonsil, appearing as if produced by the penetration of a piece of tobacco-pipe; and there was also discovered a cicatrix on each side of the tongue, marking the situation of the former wounds. A portion of the extreme end of the pipe, two inches and a half in length, was found imbedded within the substance of the tongue. The extraneous body could not possibly be seen, or even felt from within the mouth. The external carotid and all its branches, and the internal for more than an inch from its origin, were perfect: even the lingual artery had escaped injury. There was a small coagulum, mixed with mucus, in the stomach; and a firm dark clot of blood was found, extending the whole length of the respiratory tube, filling even the smallest divisions of the bronchi, although no traces could be discovered from whence the blood issued. The lungs were in a remarkable state of emphysema.

It is to be regretted that the carotid artery was not tied in this case. But the circumstances were so peculiar that hesitation in regard to the treatment may very well be excused. The case is certainly an interesting one, and may serve as as indication of what is most advisable, should a similar one occur hereafter.

D. *Oxalate of Lime Crystallized on the Surface of a Calculus.*—In the case of a boy, aged seven years, who had laboured under symptoms of stone for six years, and was operated on by Mr. Cooper, the stone was covered with crystals. The dimensions of the calculus were—length 1 inch; thickness $\frac{9}{12}$ths of an inch —breadth $\frac{11}{16}$ths of an inch. Dr. G.D. Rees examined it, and reported that:—The surface of the calculus is thickly studded with bright transparent crystals, which are deposited on a dense semi-crystalline layer of calculous matter. From the application of chemical re-agents, on which we need not dwell, he concludes:—

"We thus prove the existence of an organic acid in the crystals; which is combined with lime, and yields the carbonate of that base on being heated to redness. It having been proved that lithic acid did not exist in these crystals, and the only other organic acid which is detected in urinary concretions being the oxalic, I could not but conclude that, in all probability, oxalate of lime had assumed its crystalline form on the surface of this calculus; and, on continuing my examination, this curious fact was placed beyond a doubt. The acid was procured in combination with the oxide of silver, by precipitation of the nitrate of that base: the precipitate was white, deflagrated on the application of heat, and underwent all the re-actions of oxalate of silver. The solution of sulphate of lime is precipitated by the acid procured from these crystals, when digested in a weak solution of potash which has been allowed to become partially carbonated. The precaution of neutralizing the liquor is necessary in this experiment.

The semi-crystalline layer, on which the crystals are deposited, is composed of oxalate of lime, with a minute proportion of phosphate of lime.

The third layer from the surface consists of carbonate and phosphate of lime, with lithic acid.

It being impossible to saw this calculus without destroying the crystals, the nucleus and deep layers are not examined."

E. *Popliteal Aneurysm—Second operation.*—Signor Marani, aged 30, consulted Mr. Cooper, on account of a small aneurysmal tumor, situated in the lower part of the left popliteal space. It appeared that in the preceding August he had had the femoral artery tied for the aneurysm by Mr. George Greeves, of Manchester. The swelling never subsided, a small pulsating tumor always remaining. Mr. Greeves, in a letter to Mr. Cooper, observed :—

"There were some peculiarities in the case, which I ought to mention : one was, the early return of pulsation in the tumor, which occurred within twelve hours after the operation ; although, when the ligature was tightened during the operation, the pulsation ceased entirely, and the sac instantly became almost imperceptible ; proving, I think, that it could contain few or no coagula. Another peculiarity was, that the circulation in the limb below the ligature became almost immediately re-established : the temperature fell but little at first; and afterwards, never rose above the natural standard. Sensation also was far less interfered with than I have observed it to be in other cases ; and I have positively seen more disturbance of the circulation in the hand and fore-arm, from the tightness of a bandage after venesection.

That the femoral artery was tied, I have no doubt whatever: and Mr. Hunt, my colleague, who is an excellent anatomist, as well as Mr. Wilson, my assistant, declare, that if ever they saw a ligature put upon an artery, it was in this case. I can only conceive two modes of explaining the return of the disease ; namely, extraordinarily free anastomoses, or an irregular distribution of the vessels of the thigh. To the former opinion, most of my friends are inclined ; but Mr. Cusack of Dublin, who saw my patient when Signor Marani was obliged to make a visit to Dublin, leaned to the latter."

On a close examination of the limb, Mr. Cooper discovered, in the course of the cicatrix, the pulsation of a vessel, but certainly not of a healthy or natural-sized femoral artery ; nor did any degree of pressure upon this trunk or branch command the pulsation of the aneurysmal tumor. In the lower third of the thigh, just where the femoral artery perforates the tendon of the adductor-magnus muscle, the pulsation of the artery was very perceptible ; and pressure here immediately stopped the flow of blood into the tumor. It was in this situation, that Mr. C. proposed to tie the artery a second time. This he did with some little difficulty, tracking the vessel to the popliteal space. Immediately the ligature was applied to the artery, the pulsation in the tumor ceased, but in a few minutes became again as distinct as before the operation ; no other artery, however, could be discovered.

Some febrile excitement followed the operation. On the 20th, bloodletting was requisite, as was its repetition on the 21st. On the latter day, the pulsation in the tumor had entirely ceased, although every other tangible artery in the body pulsated strongly. The sac was quite empty, as if no coagula had formed in it. On the 26th, the compress was removed from the aneurysmal tumor, and not the least pulsation was discoverable ; yet the sac seemed to be filled with fluid blood, which could be pressed out of it. The compression was, therefore, reapplied with more force than before.

On the 22nd day after the operation, the ligature separated from the artery. He remained in bed two days after this, when the wound was found to be nearly healed. He was now wheeled into an adjoining room on a sofa, an exertion which, for two or three days he supported well enough. On the fourth day he complained of some burning sensation in the cicatrix from the wound of the first operation ; and, upon examination, a considerable blush of inflammation was perceptible upon the upper part of the thigh. A large poultice was applied ; and, in a few hours, the whole length of the cicatrix gave way, allowing the escape of a considerable quantity of matter. The discharge, however, continued but for a few days ; and upon the application of a dossil of lint, and strips of adhesive plaster, assisted by the administration of a generous diet, in less than a week the wound had again perfectly healed.

Mr. Cooper subsequently saw Signor Marani twice, the latter time in consultation with Sir Astley Cooper. The patient suffered from an inordinate action of the heart and arteries, from the slightest mental excitement. At Sir Astley's suggestion, the muriated tincture of iron was prescribed. In a few days the arterial system became calm, and the patient was enabled to go to the sea-side. In a fortnight, he returned to town perfectly well.

The following is Mr. Cooper's suggestion respecting the actual condition of the vessels in this interesting case.

"If a considerable unusual branch had been given off by one of the perforating vessels of the profunda artery, and had taken its course at the back part of the thigh, so as to enter the popliteal artery in the ham, immediately above the sac, it would necessarily occur, that the application of a ligature upon the femoral artery, below where the profunda is given off, would not prevent the flow of blood into the aneurysmal tumor; and that, therefore, the second operation became necessary. Such a distribution, accompanied also by the obliteration of the femoral in consequence of the operation of Mr. Greeves, sufficiently accounts for the circumstance of my not meeting with the artery in the usual situation of perforating the triceps muscle ; while the pulsation in the cicatrix of the original wound might have occurred, either from the enlargement of some muscular branch, or from a high division of the anastomoticus magnus. This explanation will also accord with the fact, that the deeper dissection into the popliteal space, during my operation, led me to the main trunk supplying the aneurysmal sac ; while the pulsation, which returned for a time, shews that the ligature was placed above, and so near the abnormal communicating artery, as to lead ultimately to its obliteration : then, and not till then, the pulsation in the tumor ceased, and a cure of the disease was effected."

---

### ST. GEORGE'S HOSPITAL.

This extensive and well conducted Hospital presents annually wide opportunities to its officers and students for the observation of facts. Amongst those officers, Sir Benjamin Brodie has ever been distinguished for his professional zeal. Oppressed, we may say, with the most extensive private practice which even the rank and wealth of a Metropolis gives, he is yet to be found punctually at his post, delivering a regular and extended series of clinical lectures, and giving to the students, who naturally crowd around him, that practical knowledge which familiar conversation and oral instruction can convey. This is as it should be.

Sir Benjamin Brodie's clinical lectures are published in an authentic form in our contemporary, the Medical Gazette. This precludes a copious notice of them upon our part. But we shall select a few passages, and facts, and present them in a single group.

We trust that the clinical reports from such a Hospital as St. George's, will be more extended and more systematic than they are at present. There is no great reason for complaint on this score, it is true. From no Hospital in London, perhaps, scarcely even excepting Guy's, are there more reports. But we should wish to see them systematised, and by means of the weekly and the quarterly journals, by frequent and by more elaborate modes of publication, we should desire to see our brethern throughout the country informed of what is going on within the walls of so noble an institution as the New Hospital is.

### 1. Division of the Tendo Achillis. Is it a New Operation ?

The next patient brought in was a little boy of six years of age, whose feet had been deformed from birth ; the foot being in both cases turned in, and rotated upwards and inwards, while the toes were at the same time kept pointed by contraction of the tendo Achillis. Mr. Keate inserted a narrow knife, with the flat surface between the skin and the tendon ; then turning the edge towards the tendon, it was cut across by the knife, the heel being at the same time drawn downwards, so as to separate the divided ends from one another. An instrument was then fastened upon the foot, so as to inclose it in a strap and kind of shoe, while an iron went up the leg on each side, and was buckled round the leg just below the knee. The shoe part was so contrived as to stretch the back of the leg, and allow of its being still more extended, if necessary, afterwards. The little fellow bore both operations without a single exclamation, and hardly even moved during the time. Mr. Keate afterwards explained the case to the students, and remarked that he had lately seen or heard of this operation having been revived as a new discovery, but that he had often seen it done, and as long as *five-and-thirty years* ago ; and that he believed it generally succeeded very well.—*Med. Gaz.*

The report is imperfect. It would lead the reader to suppose that the operation now so frequently performed was formerly very common and succeeded well. Then, why, the reader asks, had it fallen into disuse ? Mr. Keate, we believe, said that he saw it performed many years ago. He did not say that he saw it often performed ; indeed we have reason to think that he did not. But the operation that Mr. Keate saw was *not* the operation now in use—for, in the former, the skin was divided as well as the tendon, and in the present operation it is not divided. This makes a vast difference.

### 2. Treatment of Varicose Veins of the Leg.

First, says Sir Benjamin Brodie, enjoin rest and the horizontal posture.

Secondly, you may, if there is inflammation, bleed in the saphena vein.

Thirdly, leeches may be requisite. Apply them not *to* the inflamed part, but at a little distance *from* it.

Fourthly, the skin being excoriated, some application may be necessary. The zinc ointment or calamine cerate answers very well ; but we use, in the hospital, a preparation known with us by the name of compound chalk ointment, which is much preferable. It is, if am not mistaken, now introduced into the Pharmacopœia under the name of Ung. plumbi compositum. It is an excellent application in these and other cases where the surface of the cutis is deprived of the cuticle. This ointment was invented by Dr. Kirkland, a celebrated practitioner many years ago in Leicestershire, and I believe it was commonly known under the name of Kirkland's neutral cerate. It is composed of diachylon plaster, olive oil, chalk, and distilled vinegar. How it should have ever entered into any man's head to make such a composition as this I do not know, but the composition having been invented I must say it is a very useful one. The ointment should be spread on linen rag, and applied in stripes round the leg, each stripe over-lapping the one below. In some cases, in addition to the use of chalk ointment, you will find advantage from washing the surface with a weak solution of nitrate of silver, in the proportion of two or three grains to an ounce of rose water. A strong solution would here be improper, but a weak solution is very useful.

Fifthly, when inflammation and swelling are reduced, pressure is requisite. The pressure of a common roller will do a great deal of good, and formerly nothing else was recommended. But we find, now, that in cases of varicose ulcer, as in cases of indolent ulcer of the leg, you may very much assist the common roller by the addition of other means. One very good way of making pressure on a varicose ulcer is to interpose between it and the bandage a piece of sheet lead, such as is used in anatomical museums for covering preparations. The lead should be made quite smooth, and larger than the ulcer, extending some way beyond its margin. This makes a very uniform pressure, and really does very well. But for the most part we are in the habit of using pressure by means of plaster applied in a circular manner round the limb. It is common to employ stripes of linen spread with soap or adhesive plaster, but I own that I very much prefer diachylon plaster, for both soap plaster and adhesive plaster will frequently irritate the skin, and bring on inflammation and pustules, but diachylon plaster scarcely ever produces this effect.

In the first place the stripes should be applied round the limb, the two ends crossing each other in front, the application beginning below the ulcer, and extending some way above it. Each of the stripes ought to overlap the one below by one half of its diameter. Thus every part has a double piece of plaster over it, and you secure more equal pressure than you could otherwise obtain. It is of great consequence that the plaster should be tight enough to give comfortable support to the limb, and at the same time not so tight as to make the limb swell below; for if it does produce this effect, it is very likely that it will bring on a sloughing of the sore. The plasters ought to make uniform pressure—that is, the pressure should be equal throughout; or if there be any difference in the degree of pressure, it ought to be greater below than above. If you do not attend to this point, the plaster above operates as a tight garter, and makes the parts below swell.

When you apply the plaster, it should always be with the heel raised, the patient lying flat on his back, so that the vessels of the leg may be emptied of their blood. The same plan should be adopted when the plaster is taken off. If the leg be hanging down at the time the plaster is applied, the veins are full of blood, and the plaster becomes too loose as soon as the patient puts his leg up.

Frequently, the veins just above the heel, and behind the ankles are swelled. *Then* some stripes of plaster should be applied round the lower part of the heel, extending upwards in a longitudinal direction on each side of the leg. Let these be held firmly on while you apply the circular stripes over them, in order to keep them in their place. In this case also, in the application of the bandage, you ought to pursue the same course: a longitudinal bandage, extending under the heel and up each side of the leg, should be applied first, and this covered by a circular bandage afterwards. These may appear matters of little importance, but a great deal of your success in practice will depend on attention to such minutiæ.

Do not, says Sir Benjamin, apply dressing on lint to the ulcer. The plaster itself is the best application.

Sixthly. Sir Benjamin does *not* recommend ligature or division of the vein. He has seen serious, even fatal symptoms from both, and he has *not* seen such benefit as to warrant either.

Seventhly. Sir Benjamin thinks it right in some cases to divide, not the great venous trunk, but the vein or veins returning the blood from some particularly enlarged cluster to that trunk. Supposing, says he, I intend to cure a particular cluster of veins, I use a sharp-pointed bistoury, which cuts, not like a common bistoury, on the concave, but on the convex edge. I puncture the skin with this instrument on one side of the varicose cluster; I carry the blade under the skin, between it and the varicose veins, over to the other side of the cluster; and having carefully performed this part of the operation, the skin over it remaining entire, except where the first puncture was made, I turn the edge of the instrument backwards, and drawing it out, cut across the cluster. A good deal of hæmorrhage follows, but the pressure of a compress commands it, and a bandage is applied afterwards. The wound, in most instances, heals by the first intention. But he admits that there are few cases only in which it is worth the patient's while to submit even to this.—*Med. Gazette.*

### 3. Tables and Calculations relating to Rheumatism. By Dr. R. M'Leod.

*Tables and Inferences drawn from* 150 *Cases of Acute, Chronic, and Capsular Rheumatism, treated at St. George's Hospital.*

A. *Liability of different Ages.*

*Cases of acute rheumatism,* Males, 55 ; Females, 30. } 85

| Ages. | Males. | Females. | Total. |
|---|---|---|---|
| 10 to 15 | 1 | 0 | 1 |
| 15 to 20 | 9 | 10 | 19 |
| 20 to 25 | 9 | 4 | 13 |
| 25 to 30 | 15 | 7 | 22 |
| 30 to 35 | 8 | 3 | 11 |
| 35 to 40 | 8 | 0 | 8 |
| 40 to 45 | 3 | 2 | 5 |
| 45 to 50 | 1 | 0 | 1 |
| 50 to 55 | 0 | 2 | 2 |
| 55 to 60 | 1 | 2 | 3 |

From this table it would appear that in both sexes from 15 to 30 is the time of life most obnoxious to acute rheumatism, 54 out of 85 cases having occurred within the period specified.

B. *Duration of Complaint.*

*Periods during which the Cases of Acute Rheumatism were under treatment.*

| Cured. | | Died. |
|---|---|---|
| In 5 days ..... | 1 | Of pericarditis, 2; viz. at the end of three days, 1; at the end of three months, of a second attack, 1. |
| In 8 days ..... | 6 | |
| In 10 days..... | 4 | |
| In 12 days..... | 4 | |
| In 2 weeks.... | 7 | |
| In 3 weeks.... | 22 | |
| In 4 weeks.... | 14 | |
| In 5 weeks.... | 3 | |
| In 6 weeks.... | 9 | [Date of discharge not recorded in 3.] |
| In 8 weeks.... | 6 | |
| In 10 weeks .. | 1 | |
| In 16 weeks ... | 2 | |

It thus appears that 58 out of 82 cases were discharged cured within a month; and when it is kept in mind that it is the custom of the hospital not to send out patients who have had acute rheumatism as soon as they cease to complain of pain, we may safely assert that the number specified were cured in little if at all exceeding three weeks. But 44, or rather more than one-half, were actually discharged within three weeks; and certainly the great majority of these were free from complaint at the end of a fortnight.

C. *Occurrence of Pericarditis.*

Of the 85 cases of acute rheumatism above alluded to, the heart was implicated in 18, or rather more than one-fifth.

But of the total number of patients, 55 were males, and only 30 females. Now of the men not more than seven had pericarditis, whereas of the women, eleven, or about one in three, had symptoms indicating that condition.

Of the eighteen cases in which the heart was affected, there was only one in which the pain of the limbs was simultaneously so alleviated as to give the least colouring to the idea of metastasis. In seventeen of the cases alluded to, the heart-affection was preceded by rheumatism of the limbs; in one case the pericarditis came on first, and the rheumatism of the extremities subsequently, but within twenty-four hours.

The following are the ages of those who had rheumatic pericarditis :—

| Age. | Males. | Females. | Total. |
|---|---|---|---|
| 10 to 15 | 1 | 0 | 1 |
| 15 to 20 | 2 | 4 | 6 |
| 20 to 25 | 2 | 4 | 6 |
| 25 to 30 | 1 | 2 | 3 |
| 30 to 35 | 1 | 0 | 1 |
| 55 to 60 | 0 | 1 | 1 |
| | 7 | 11 | 18 |

Dr. M'Cleod is inclined to think, from his dispensary experience, that rheumatic pericarditis is more frequent in the young, than in those more advanced in years.

Of those referred to in the preceding table of cases of acute rheumatism, but one was under 15 years, and he had pericarditis. Of cases in subjects between 15 and 20, there were twenty; and of them six had pericarditis. Between 20 and 25 there were thirteen; and of them six had pericarditis. After this the proportion diminishes, for while there were twenty-two cases of acute rheumatism between the ages of 25 and 30, yet they give but three cases of pericarditis; 30 to 35 give ten cases of rheumatism, and one of pericarditis; 35 to 40 give eight cases of rheumatism, and none of heart-affection.

D. We pass over chronic rheumatism, and pause at *Synovial Rheumatism.* Of this there were 23 cases—16 in males—7 in females.

| Ages. | Males. | Females. | Total. |
|---|---|---|---|
| 15 to 20 | 1 | 0 | 1 |
| 20 to 25 | 1 | 1 | 2 |
| 25 to 30 | 2 | 1 | 3 |
| 30 to 35 | 3 | 2 | 5 |
| 35 to 40 | 1 | 1 | 2 |
| 30 to 45 | 2 | 1 | 3 |
| 45 to 50 | 3 | 0 | 3 |
| 50 to 55 | 2 | 0 | 2 |
| 55 to 60 | 1 | 0 | 1 |
| 60 to 65 | 0 | 1 | 1 |

According to this table, capsular rheumatism would seem to be considerably more prevalent among men than women, and much more equally diffused over different periods of adult age, than the form of the disease first described.

*Periods during which the Cases of Capsular Rheumatism were under Treatment.*

| | | | | | |
|---|---|---|---|---|---|
| 3 weeks.... | 3 | 7 weeks.... | 2 | 3 months.... | 2 |
| 4 do....... | 3 | 8 do....... | 5 | 4 do......... | 1 |
| 6 do....... | 4 | 10 do....... | 2 | 6 do......... | 1 |

The most remarkable circumstance in this table is the length of time during which the patients were under treatment, as compared to the other forms of the disease. Of the cases of acute rheumatism, as we have seen, more than one-half were discharged within three weeks; whereas here the number discharged in the same period scarcely exceeded 1-8th. Again, of the acute cases, less than 1-9th remained so long as two months in the hospital; whereas, of the synovial cases, within a fraction of one-half were in the hospital two months and upwards.

Of the cases, three were fatal,—viz. one from suppuration in the joints at the end of two months; one from pleurisy, at the end of seven weeks: and one from inflammation of the encephalon, at the end of three months.

The cases of periosteal rheumatism which have occurred within the period in which the above relates, amounts to 10; and those of arthritic or rheumatic pain, following the course of particular nerves (all of the lower extremities), to 7;—numbers too small on which to hazard any general inferences.

